# Endofungal Bacterial Microbiota Promotes the Absorption of Chelated Inorganic Phosphorus by Host Pine through the Ectomycorrhizal System

**DOI:** 10.1128/spectrum.00162-23

**Published:** 2023-07-05

**Authors:** Ai-Yue Zhang, Mei-Ling Zhang, Jia-Le Zhu, Yan Mei, Fang-Ji Xu, Hong-Yan Bai, Kai Sun, Wei Zhang, Chuan-Chao Dai, Yong Jia

**Affiliations:** a Jiangsu Key Laboratory for Microbes and Functional Genomics, Jiangsu Engineering and Technology Research Center for Industrialization of Microbial Resources, College of Life Sciences, Nanjing Normal University, Nanjing, China; b Institute of Crop Germplasm Resources, Shandong Academy of Agricultural Sciences (SAAS), Jinan, China; University of Mississippi

**Keywords:** ectomycorrhizal fungi, endofungal bacteria, interaction, chelated inorganic phosphorus, organic acid

## Abstract

Ectomycorrhizal fungi play an irreplaceable role in phosphorus cycling. However, ectomycorrhizal fungi have a limited ability to dissolve chelated inorganic phosphorus, which is the main component of soil phosphorus. Endofungal bacteria in ectomycorrhizal fruiting bodies are always closely related to the ecological function of ectomycorrhizal fungi. In this study, we explore endofungal bacteria in the fruiting body of *Tylopilus neofelleus* and their function during the absorption of chelated inorganic phosphorus by host pine through the ectomycorrhizal system. The results showed that the endofungal bacterial microbiota in the fruiting body of *T. neofelleus* might be related to the dissolution of chelated inorganic phosphorus in soil. The soluble phosphorus content in the combined system of *T. neofelleus* and endofungal bacteria *Bacillus* sp. strain B5 was five times higher than the sum of *T. neofelleus*-only treatment and *Bacillus* sp. strain B5-only treatment in the dissolution experiment of chelated inorganic phosphorus. The results showed that *T. neofelleus* not only promoted the proliferation of *Bacillus* sp. strain B5 in the combined system but also improved the expression of genes related to organic acid metabolism, as assesed by transcriptomic analysis. Lactic acid content was five times higher in the combined system than the sum of *T. neofelleus*-only treatment and *Bacillus* sp. strain B5-only treatment. Two essential genes related to lactate metabolism of *Bacillus* sp. strain B5, *gapA* and *pckA*, were significantly upregulated. Finally, in a pot experiment, we verified that *T. neofelleus* and *Bacillus* sp. strain B5 could synergistically promote the absorption of chelated inorganic phosphorus by *Pinus sylvestris* in a ternary symbiotic system.

**IMPORTANCE** Ectomycorrhizal fungi (ECMF) have a limited ability to dissolve chelated inorganic phosphorus, which is the main component of soil phosphorus. In the natural environment, the extraradical hyphae of ECMF alone may not satisfy the phosphorus demand of the plant ectomycorrhizal system. In this study, our results innovatively show that the ectomycorrhizal system might be a ternary symbiont in which ectomycorrhizal fungi might recruit endofungal bacteria that could synergistically promote the mineralization of chelated inorganic phosphorus, which ultimately promotes plant phosphorus absorption by the ectomycorrhizal system.

## INTRODUCTION

Ectomycorrhizal fungi (ECMF) are typical symbionts and play an irreplaceable role in promoting nutrient cycling in the ecosystem (1, 2). ECMF form a symbiotic interface with plant roots where ECMF receive carbon from the host plant; in return, extraradical hyphae can expand the nutrient absorption area of ECMF and thus increase the nutrient supply to host plants, especially poorly mobile phosphate ions (3–5). However, ECMF have a limited ability to dissolve chelated inorganic phosphorus, which is the main component of the soil phosphorus source (1, 6). Therefore, in the natural environment, ECMF alone may not satisfy the phosphorus demand of the plant ectomycorrhizal system.

Previous studies have shown that ECMF release certain compounds, such as trehalose, to recruit specific bacteria that could promote the growth of ectomycorrhizal hyphae and ectomycorrhizal formation from soil bacterial microbiota (7–11), and some of them are also closely related to the mineral release in soil by releasing various enzymes and organic acids (e.g., phosphorus) (12, 13). Moreover, these recruited bacteria may adhere to the mycelia and eventually enter the fruiting bodies to form unique endofungal bacterial microbiota during the formation of the ectomycorrhizal fruiting bodies (14–18). A previous study showed that bacterial taxa involved in the decomposition of organic material were relatively more abundant in saprotrophic fruiting bodies, whereas those involved in the release of minerals were relatively more enriched in ectomycorrhizal fruiting bodies (16). Consequently, there should be some bacteria able to dissolve chelated inorganic phosphorus in the ectomycorrhizal fruiting body, which could cooperate with ECMF to synergistically promote the absorption of chelated inorganic phosphorus of the host plant through the ectomycorrhizal system.

*Tylopilus neofelleus* (MZ726385.1, family *Boletaceae*, genus *Tylopilus*) is a typical ectomycorrhizal fungus that can form ectomycorrhizae with pine trees and fruiting bodies. Here, we will explore the endofungal bacterial microbiota in the fruiting body of *T. neofelleus* and their function and mechanism to synergize dissolved chelated inorganic phosphorus with *T. neofelleus*. Furthermore, a pot experiment was used to verify the promoting effect of the combined system of ECMF and their endofungal bacteria on the absorption of phosphorus by pine through its ectomycorrhizal system.

## RESULTS

### Comparison of endofungal bacterial microbiota in the fruiting body and corresponding bacterial microbiota in mycosphere soil.

**FIG 1:**
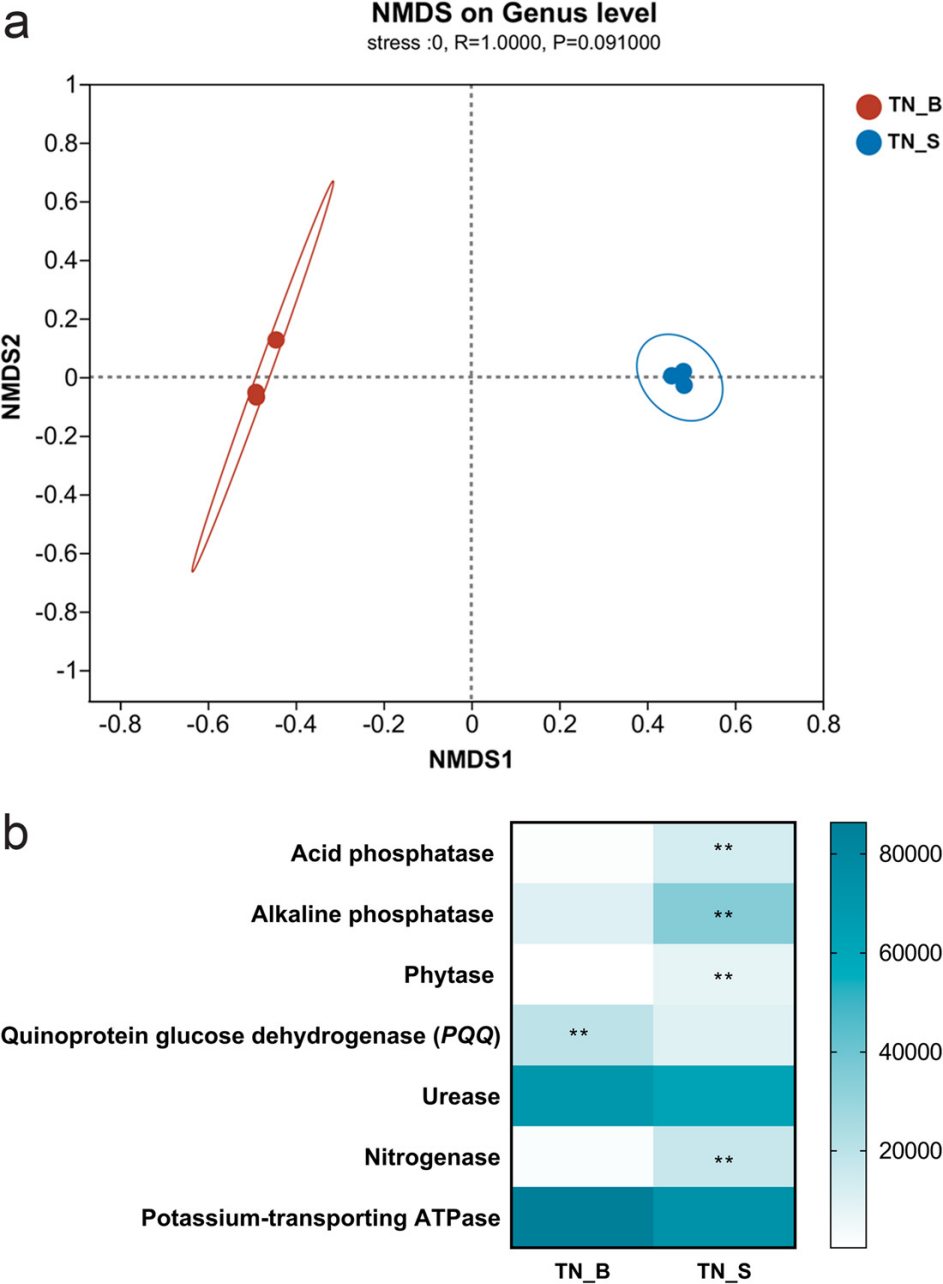
Comparison of the endofungal bacterial microbiota and mycosphere soil bacterial microbiota of *T. neofelleus*. a, Nonmetric multidimensional scaling analyses (NMDS) of Bray-Curtis distance of endofungal bacterial microbiota in the fruiting body and mycosphere soil bacterial microbiota at the genus level. b, Variation in bacterial function profiles analyzed by PICRUSt. Data were analyzed by independent sample *t* test and Mann-Whitney *U* test; **, *P* < 0.01; TN_B, endofungal bacterial microbiota of *Tylopilus neofelleus*; TN_S, mycosphere soil bacterial microbiota of *Tylopilus neofelleus*.

Differences in bacterial α diversity were analyzed between the endofungal bacterial microbiota in the *T. neofelleus* fruiting body and the corresponding bacterial microbiota in mycosphere soil. The results showed that bacterial richness was significantly different between the endofungal bacterial microbiota in the *T. neofelleus* fruiting body and the soil bacterial microbiota in mycosphere soil. The Shannon index, abundance-based coverage estimator (ACE) index, and Chao index of the endofungal bacterial microbiota were much lower than those of mycosphere soil bacterial microbiota (*P* < 0.001), while the Simpson index was much higher than that in the mycosphere soil bacterial microbiota (*P* < 0.001) (Fig. S4 in the supplemental material). *Proteobacteria* was the most abundant bacterial phylum in the *T. neofelleus* fruiting body and mycosphere soil, accounting for 94.2% and 40.48%, respectively (Fig. S5a). Moreover, *Rhizobiales* and *Burkholderiales* dominated in the fruiting body, whereas *Rhizobiales* and *Rhodospirillales* dominated in the mycosphere soil at the order level (Fig. S5b). Richness of the top 10 bacterial communities was also significantly different at the phyla and order levels (Fig. S6). Nonmetric multidimensional scaling analyses (NMDS) showed that bacterial diversity was different between the endofungal bacterial microbiota and mycosphere soil bacterial microbiota at the genus, order, and phylum levels (stress = 0, *P* = 0.091) (Fig. 1a; Fig. S5c and d). In short, our results suggest that the *T. neofelleus* fruiting body might obtain specific endofungal bacterial microbiota from the mycosphere soil bacterial microbiota.

### Chelated inorganic phosphorus dissolution ability of cultivable endofungal bacteria and *T. neofelleus*.

A previous study showed that fungi may choose endofungal bacteria that can support the functional roles of these fungi in terrestrial ecosystems, so endofungal bacteria involved in the release of minerals were relatively more enriched in the ectomycorrhizal fruiting body (16). In this study, *Rhizobiales* and *Burkholderiales* were often reported among a group of bacteria with strong mineralization abilities that accounted for a high proportion of the endofungal bacterial microbiota of *T. neofelleus* fruiting bodies (19). Moreover, abundance of the quinoprotein glucose dehydrogenase gene related to chelated inorganic dissolution of endofungal bacterial microbiota was much higher than that in the soil bacterial microbiota (*P* = 0.008) (Fig. 1b). By contrast, the abundances of alkaline phosphatase (ALP), acid phosphatase (ACP), phytase, and nitrogenase in the endofungal bacterial microbiota were much lower than those observed in the soil bacterial microbiota (*P* < 0.01) (Fig. 1b). In this study, the soluble phosphorus content in the combined treatment increased significantly compared to that in the bacteria-only treatment (*P* < 0.05) (Fig. S1). The *T. neofelleus*-only treatment also had a weak ability to dissolve chelated inorganic phosphorus (Fig. S1). Furthermore, the synergistic phosphorus dissolution abilities of Bacillus aerius strain B3, *Bacillus* sp. strain B5, and Bacillus altitudinis strain B14 with *T. neofelleus* were significantly higher than those in the other treatments. The combined system of *Bacillus* sp. strain B5 and *T. neofelleus* had the highest soluble phosphorus content in the dissolution experiment of chelated inorganic phosphorus (Fig. S1). Thus, we selected the endofungal bacteria *Bacillus* sp. strain B5 that could adhere to the mycelial surface of *T. neofelleus* to further study the dissolution characteristics and mechanism of chelated inorganic phosphorus in the combined system (Fig. S2).

### Dissolution mechanism of chelated inorganic phosphorus by the combined system.

**FIG 2:**
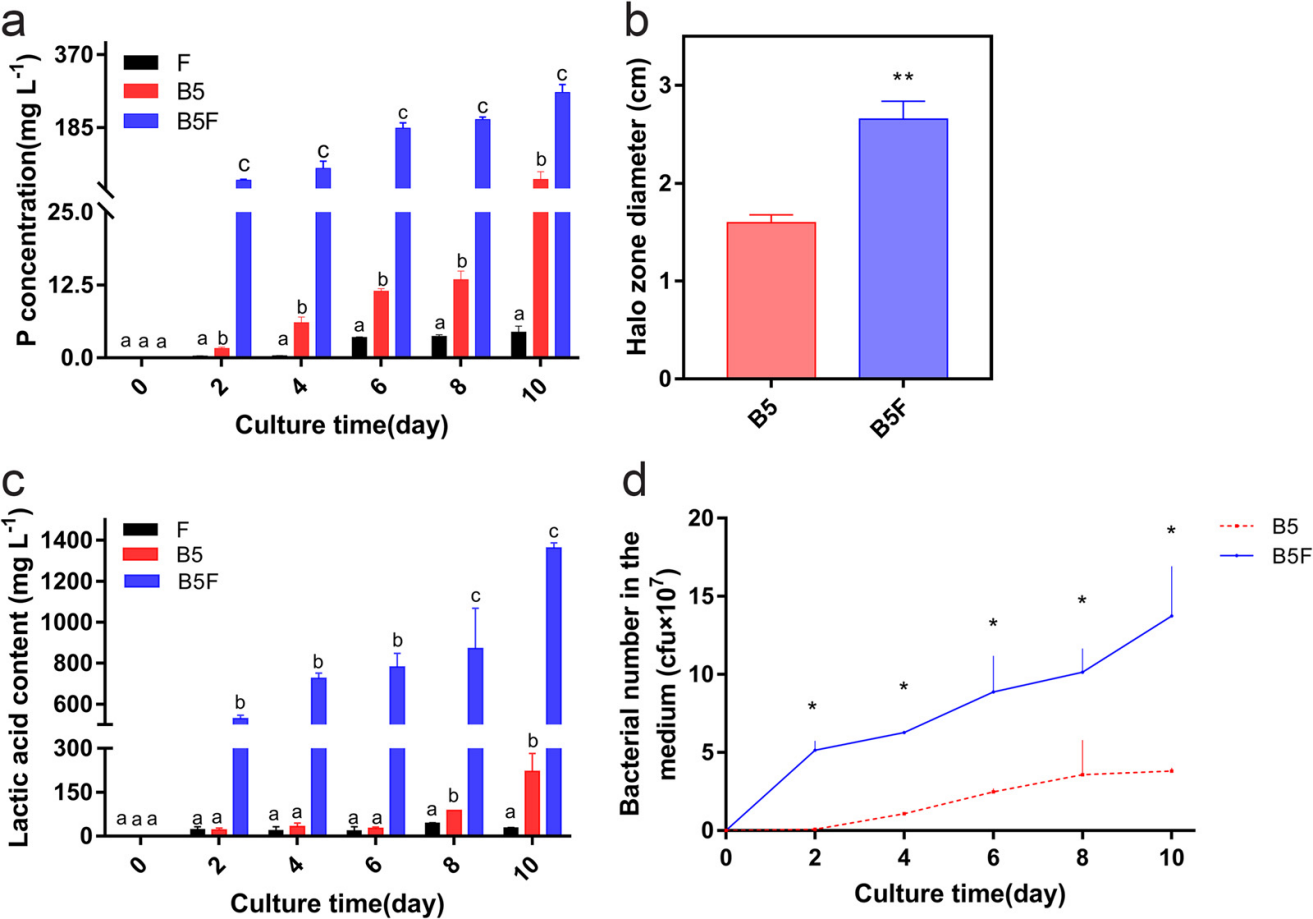
Chelated inorganic phosphorus dissolution ability of the combined system. a, Soluble phosphorus content in the culture medium. b, Bacterial phospholysis circle size on the culture plate. c, Lactic acid content in culture medium. d, Biomass of *Bacillus* sp. strain B5. Values are shown as mean ± SE of three independent replicates. Bars with different letters indicate statistically significant differences. Data in a and c were analyzed by Kruskal-Wallis analysis of variance tests with a *post hoc* Tukey and LSD multiple range test (*P* < 0.05). Data in b were analyzed by Mann-Whitney *U* test (*P* = 0.002); *, *P* < 0.05; **, *P* < 0.01; ***, *P* < 0.001. Data in d were analyzed by Mann-Whitney *U* test (*P* < 0.001); B5, *Bacillus* sp. strain B5-only treatment; F, *T. neofelleus*-only treatment; B5F, combined treatment of *T. neofelleus* and *Bacillus* sp. strain B5.

The results showed that the soluble phosphorus content was 274 mg L^−1^ in the combined system, while it was only 53.78 mg L^−1^ and 4.41 mg L^−1^ in the *Bacillus* sp. strain B5-only treatment and *T. neofelleus*-only treatment after 10 days, respectively (*P* < 0.05) (Fig. 2a). The soluble phosphorus content was significantly increased in the combined system, which was five times as much as the sum of the *Bacillus* sp. strain B5-only treatment and *T. neofelleus*-only treatment. The results of the plate experiments were also consistent with the dissolution study. The diameter of the phosphorus solubilization circle was 2.8 cm in the combined system and only 1.6 cm in the *Bacillus* sp. strain B5-only treatment (*P* = 0.002) (Fig. 2b; Fig. S7). In the dissolution study, the organic acid content was detected by high-performance liquid chromatography (HPLC). The combined treatment showed a significant increase in the concentration of lactic acid (1,361.6587 mg L^−1^) compared to the *Bacillus* sp. strain B5-only treatment (221.248 mg L^−1^) and *T. neofelleus*-only treatment (28.03 mg L^−1^; *P* < 0.05) (Fig. 2c). To investigate the mechanism of the combined system on the solubilization of chelated inorganic phosphorus, numbers of *Bacillus* sp. strain B5 cells in the culture medium were measured, and the *Bacillus* sp. strain B5 sample for RNA sequencing (RNA-seq) was also collected after 4 days of different treatments. Compared with the *Bacillus* sp. strain B5-only treatment, the amount of *Bacillus* sp. strain B5 increased significantly in the combined system at different times (*P* < 0.001) (Fig. 2d). The results showed that *T. neofelleus* promoted the growth of *Bacillus* sp. strain B5 in the combined system. Moreover, *T. neofelleus* could also regulate the organic acid metabolism of *Bacillus* sp. strain B5 in the combined system. The results showed that *Bacillus* sp. strain B5 had significant differences in the expression of 346 genes in the combined system compared with the control, including 232 genes with increased expression levels and 114 genes with decreased expression levels (Fig. S8). Furthermore, the enzyme activities of alkaline phosphatase, acid phosphatase, and phytase were measured following different treatments (Table S2). There was no significant difference in alkaline phosphatase activity or alkaline phosphatase gene expression levels between the *Bacillus* sp. strain B5-only treatment and the combined treatment (*P* > 0.05) (Fig. S9). Moreover, genomic analysis revealed the absence of acid phosphatase and phytase genes in *Bacillus* sp. strain B5. Thus, there were also no significant differences in acid phosphatase and phytase activities between the *T. neofelleus*-only treatment and the combined treatment (*P* > 0.05).

**FIG 3:**
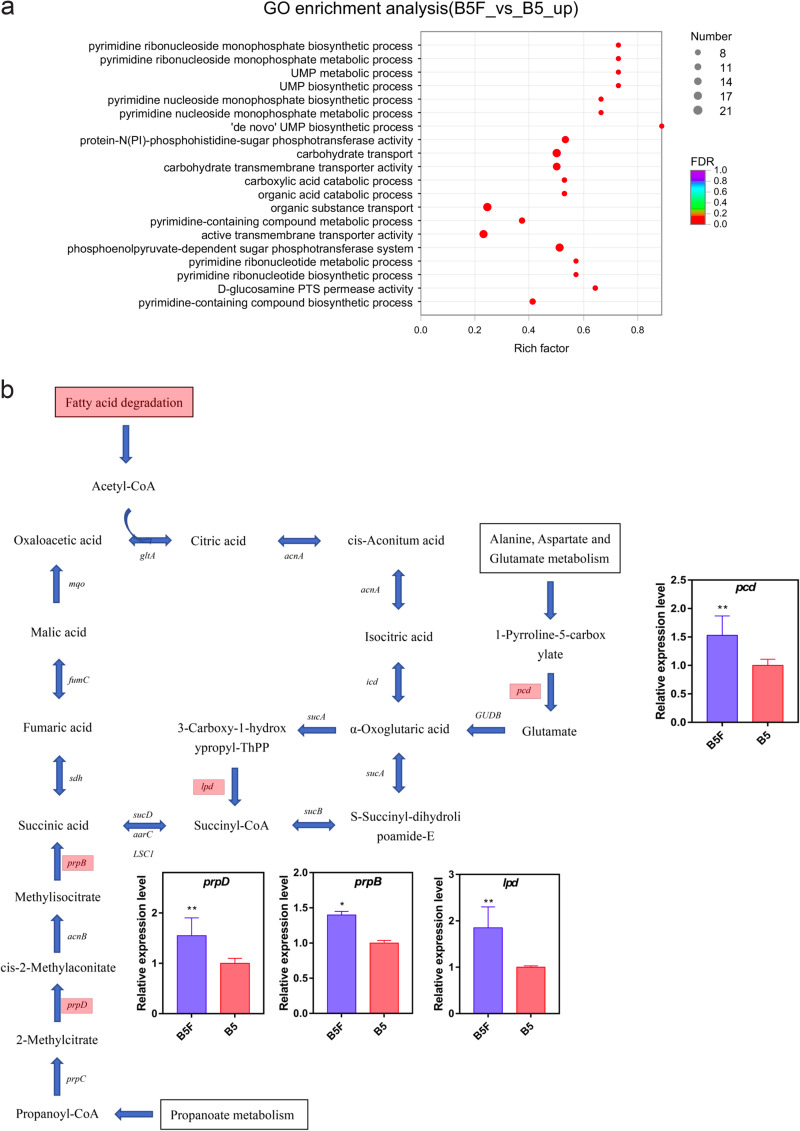

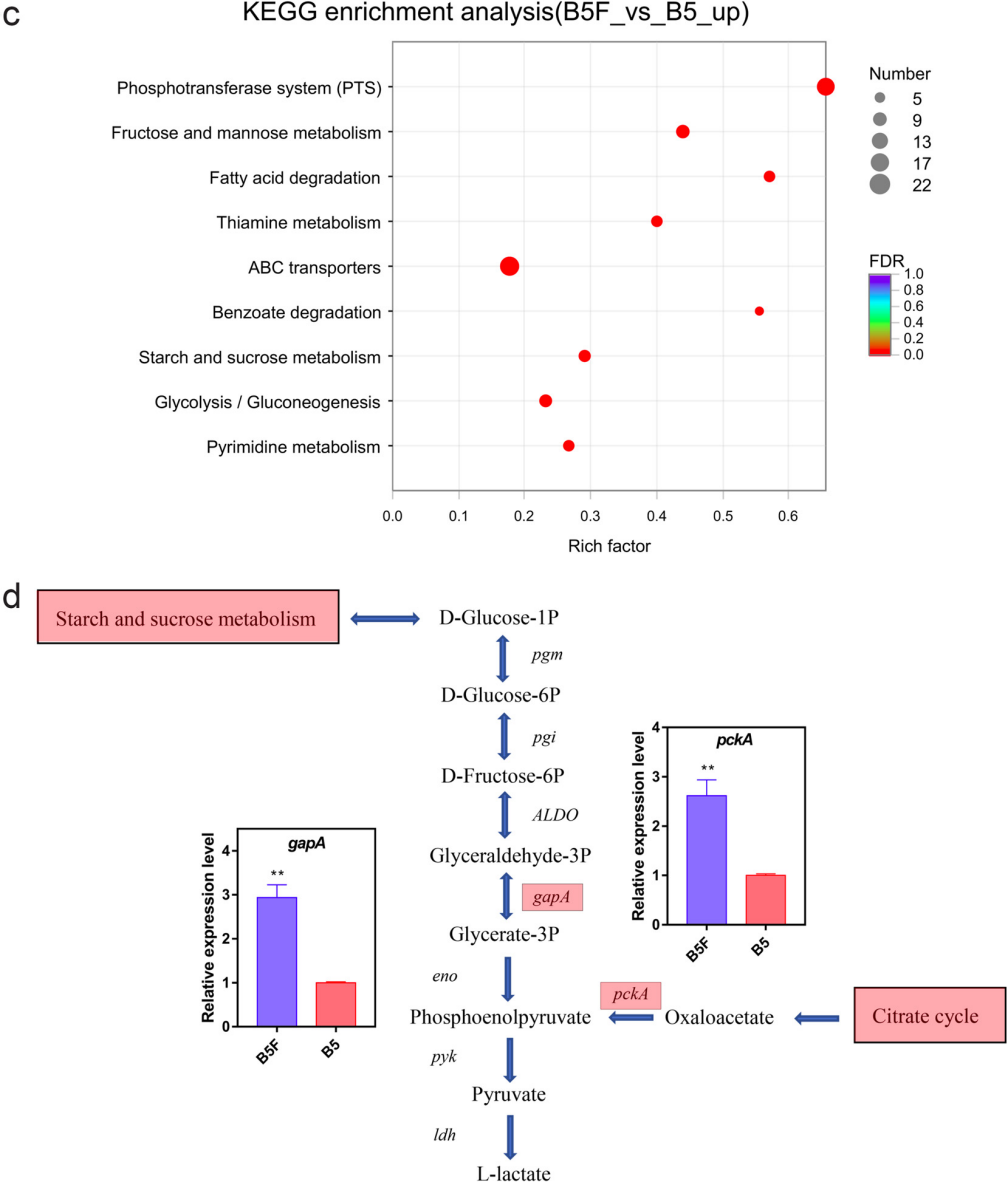
Transcriptomic analysis by RNA-seq and RT-qPCR. a, Gene Ontology (GO) analysis of differentially expressed genes of *Bacillus* sp. strain B5 between *Bacillus* sp. strain B5-only treatment and the combined treatment. b, Upregulated expressed genes related to the organic acid metabolic pathway. c, KEGG analysis of differentially expressed genes of *Bacillus* sp. strain B5 in the *Bacillus* sp. strain B5-only treatment and the combined treatment. d, Upregulated expressed genes related to the lactic acid metabolic pathway. Values are shown as mean ± SE of three independent replicates. Bars with different letters indicate statistically significant differences. Data in b were analyzed by independent sample *t* test (*prpD*, *P* = 0.001; *prpB*, *P* = 0.026) and Mann-Whitney *U* test (*lpd* and *pcd*, *P* = 0.002). Data in d were analyzed by Mann-Whitney *U* test (*gapA* and *pckA*, *P* = 0.002); B5, *Bacillus* sp. strain B5-only treatment; F, *T. neofelleus*-only treatment; B5F, combined treatment of *T. neofelleus* and *Bacillus* sp. strain B5; FDR, false-discovery rate.

Gene Ontology (GO) enrichment analysis of differentially expressed genes was then performed (Fig. S10). Most of the differentially expressed genes with predicted functional annotations were assigned to biological process, cellular component, and molecular function (Fig. S10a). Among the differentially expressed genes, the organic acid catabolic pathway was significantly upregulated in *Bacillus* sp. strain B5 in the combined treatment (Fig. 3a), in which the expression of key genes in the organic acid metabolic pathway, such as methylisocitrate lyase (*prpB*; *P* = 0.026), l-glutamate γ-semialdehyde dehydrogenase (*pcd*; *P* = 0.026), aconitate hydratase (*prpD*; *P* = 0.001), and dihydrolipoamide dehydrogenase (*lpd*; *P* = 0.002), was significantly upregulated (Fig. 3b). We then performed KEGG pathway enrichment analysis for the differentially expressed genes. KEGG pathway analysis showed that amino acid metabolism, carbohydrate metabolism, energy metabolism, metabolism of cofactors and vitamins, and membrane transport were enriched (Fig. S11). Consistently, several genes related to glycolytic metabolism were upregulated when *Bacillus* sp. strain B5 was cocultivated with *T. neofelleus* in the combined treatment (Fig. 3c). In addition, genes associated with lactate metabolism (*gapA* and *pckA*) were increased in the combined treatment (*gapA* [*P* = 0.023] and *pckA* [*P* = 0.020]) (Fig. 3d). These results suggest that *T. neofelleus* can regulate the organic acid catabolism of *Bacillus* sp. strain B5, which in turn promotes the solubilization of chelated inorganic phosphorus. Using real-time quantitative PCR (RT-qPCR), the expression of eight randomly selected genes was assessed and showed similar trends to the RNA-seq data (*P* < 0.05) (Fig. S12).

### Plant growth properties and phosphorus content in the pot experiment.

**FIG 4:**
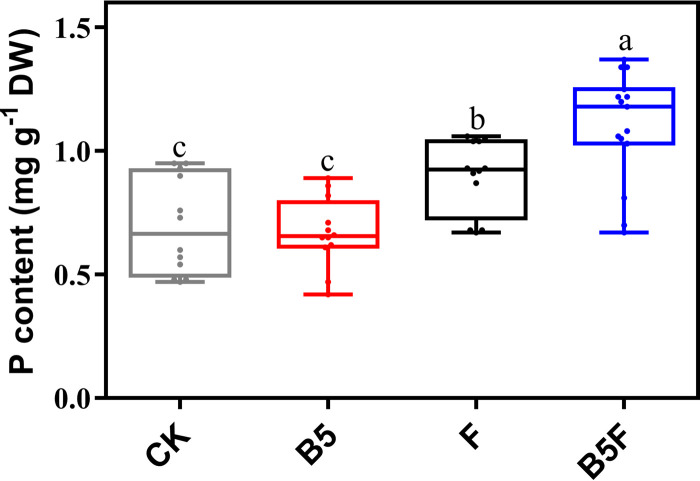
Phosphorus content of *P. sylvestris*. Values are shown as mean ± SE of six independent replicates. Bars with different letters indicate statistically significant differences. Data were analyzed by one-way analysis of variance and Tukey’s multiple range test (*P* < 0.05); CK, control treatment; B5, *Bacillus* sp. strain B5-only treatment; F, *T. neofelleus*-only treatment; B5F, the combined treatment of *T. neofelleus* and *Bacillus* sp. strain B5.

*Pinus sylvestris* seedlings could form an ectomycorrhizal structure with *T. neofelleus* in the *T. neofelleus*-only treatment and the combined treatment, including root binary branches, mantles, and Hartig nets (Fig. S13). There was no significant difference in the ectomycorrhizal colonization rates of *P. sylvestris* between the *T. neofelleus*-only treatment and the combined treatment (*P* > 0.05) (Table S3). Moreover, the phosphorus content in the plant was significantly higher in the combined treatment and *T. neofelleus*-only treatment than in the *Bacillus* sp. strain B5-only treatment and the control treatment in the pot experiment (*P* < 0.05) (Fig. 4). The plant phosphorus content was 58% higher in the combined treatment than in the control treatment (*P* < 0.001) (Fig. 4). We also tested the content of available phosphorus in three vermiculite areas of the pot. In the plant and buffer compartments, the available phosphorus content was significantly higher in the combined treatment and the *T. neofelleus*-only treatment than in the *Bacillus* sp. strain B5-only treatment and the control treatment (Fig. S14a and b). However, there was no significant difference in the available phosphorus content among the different treatments in the mycelial compartment (*P* > 0.05) (Fig. S14c). The total phosphorus and available phosphorus in vermiculite showed the same trend (Fig. S14d to f). These results indicate that the combined treatment of ECMF and endofungal bacteria significantly promotes the uptake of chelated inorganic phosphorus by *P. sylvestris* through the ectomycorrhizal system. Moreover, the pH values in the mycelial compartment of the other three treatments were slightly lower than that observed in the control treatment (*P* < 0.05) (Fig. S15). *Pinus sylvestris* seedlings under the combined treatment were significantly taller than those under the control treatment in terms of shoot length, root length, and dry weight (*P* < 0.05) (Fig. S16). By contrast, the number of lateral roots was not significantly different at 90 days after planting (*P* > 0.05) (Fig. S16).

## DISCUSSION

### Endofungal bacterial microbiota richness and structure in the fungal fruiting body.

In this study, we found that there was a difference in structure between the endofungal bacterial microbiota in fruiting bodies and the corresponding mycosphere soil bacterial microbiota, as assessed by Illumina high-throughput sequencing and NMDS analysis. The endofungal bacterial microbiota richness was significantly less than that of the mycosphere soil microbiota, consistent with previous research results (14, 15). Although the endofungal bacterial microbiota of ectomycorrhizal fungi was mainly recruited from the mycosphere soil bacterial microbiota, they had different functions (14, 16). Previous studies also showed that bacterial taxa involved in the decomposition of organic material were relatively more abundant in the saprotrophic fruiting body, whereas those involved in the release of minerals were relatively more enriched in the ectomycorrhizal fruiting body (16). In our study, *Rhizobiales* and *Burkholderiales*, which had strong mineralization abilities, were dominant in the endofungal bacterial microbiota in the fruiting body (19, 20). Moreover, the molecular functions of bacterial microbiota from each sample were predicted by PICRUSt based on KEGG databases. The results showed that the abundance of the quinoprotein glucose dehydrogenase gene related to the chelated inorganic dissolution of endofungal bacterial microbiota was much higher than that of the mycosphere soil bacterial microbiota (*P* < 0.05) (Fig. 1b). Furthermore, most isolated cultivable endofungal bacteria had phosphate dissolution abilities on chelated inorganic phosphorus compared with the weak dissolution ability of ECMF in this study (*P* < 0.05) (Fig. S1). A previous study also showed that the endofungal bacterial microbiota in ectomycorrhizal fungi was associated with chelated state mineral dissolution functions (16). Moreover, mycorrhizal fungi could also alter the cycling of soil nutrients by influencing the intermycelial microbial composition, such as C, N, and P (21, 22). Thus, the results indicated that ECMF might recruit endofungal bacteria with chelated inorganic phosphorus dissolution abilities from soil bacterial microbiota to support the functional roles of ECMF in terrestrial ecosystems.

### Dissolution of chelated inorganic phosphorus by the combined system of endofungal bacterial microbiota and ECMF.

In our study, the dissolution ability on chelated inorganic phosphorus was synergistically promoted in the combined treatment of *T. neofelleus* and *Bacillus* sp. strain B5 (*P* < 0.05) (Fig. 2a), which might be because *T. neofelleus* could promote bacterial solubilization of chelated inorganic phosphorus by regulating organic acid metabolism of *Bacillus* sp. strain B5 (*P* < 0.05) (Fig. 2c) and promoting *Bacillus* sp. strain B5 proliferation in the combined system (*P* < 0.05) (Fig. 2d). Previous studies have shown that ectomycorrhizal fungi that are thiamine synthesis deficient need to coexist with soil bacteria that can produce thiamine to promote their own mycelial growth (23). The soluble phosphorus content was also significantly increased in the symbiosis of arbuscular mycorrhizal fungi (AMF) and phosphate-solubilizing bacteria (PSB) because AMF could promote the synthesis and secretion of phytase and phosphatase by PSB (24). Moreover, the dissolution of chelated inorganic phosphorus was mainly achieved by the secretion of organic acids, while the types of organic acids produced by different bacteria were diverse. *Bacillus* sp. is often related to the secretion of organic acids, including lactic acid, citric acid, propionic acid, and gluconic acid (25). Our results showed that the lactic acid content of *Bacillus* sp. strain B5 was significantly increased in the combined system (*P* < 0.05) (Fig. 2c). The expression levels of two essential genes (*gapA* and *pckA*) associated with lactate metabolism were significantly upregulated in *Bacillus* sp. strain B5 in the combined system (*gapA* and *pckA*, *P* = 0.002) (Fig. 3d). Previous studies have also shown that acid phosphatase genes of the PSB Rahnella aquatilis could be regulated by fructose secreted by the AMF *Rhizophagus irregularis* (26). In our study, the biomass of *Bacillus* sp. strain B5 was also increased when coinoculated with *T. neofelleus*, which indicated that *T. neofelleus* might promote the growth of *Bacillus* sp. strain B5 in the combined system (*P* < 0.05) (Fig. 2d). Previous studies have also shown that fungi can support different carbon sources and energy for endofungal bacteria in the fruiting body (16, 27). Thus, the results suggest that ectomycorrhizal fungi may synergistically promote the dissolution of chelated inorganic phosphorus by regulating the changes in growth and metabolism-related genes of the endofungal bacteria they recruit.

### *T. neofelleus* and *Bacillus* sp. strain B5 synergistically promote chelated inorganic phosphorus uptake by *P. sylvestris*.

In the pot experiment, the results showed that the synergistic effect of the endofungal bacterial *Bacillus* sp. strain B5 and *T. neofelleus* promoted the absorption of chelated inorganic phosphorus by *P. sylvestris* through the ectomycorrhizal system. In the buffer compartment of the pot, the content of available phosphorus was lower, and the total phosphorus was significantly higher in the *T. neofelleus*-only treatment and the combined treatment than in the other treatments (*P* < 0.05) (Fig. S14b and e), which indicated that the phosphorus might mainly be transported to plants through ectomycorrhizal hyphae from the hyphal compartment. Previous studies have mainly shown that the synergistic interaction between AMF and PSB could also enhance phosphorous uptake in plants through arbuscular mycorrhizal hyphae (24, 28, 29). In the combined treatment, our results suggest that the endofungal bacterial microbiota might help ECMF mineralize chelated inorganic phosphorus and promote phosphorus absorption by ECMF, which promoted plant phosphorus absorption by the ectomycorrhizal system. However, there were no significant differences in the height and weight of *P. sylvestris* between the combined treatment and other treatments, which might be due to the short cultivation time of the plant (Fig. S16). Moreover, a previous study suggested that AMF combined with trifoliate orange (*Poncirus trifoliata* L. Raf.) seedlings could improve plant resistance to stress by promoting the growth of plant roots (30). In this study, the number of lateral roots of *P. sylvestris* also increased significantly in *T. neofelleus*-only treatment, whereas there was no significant difference between the combined treatment and the control treatment (Fig. S16c). The results suggest that ECMF might promote the growth of the plant lateral roots only under low-phosphorus stress conditions (30), while low-phosphorus stress in the combined treatment might be alleviated.

Overall, the results showed that there were some endofungal bacteria in the ectomycorrhizal fruiting body that could regulate the metabolism of organic acids by ECMF to synergistically promote the dissolution of chelated inorganic phosphorus, thus increasing the content of phosphorus absorbed by plants through the ectomycorrhizal system. The ectomycorrhizal system might be a ternary symbiont, in which ectomycorrhizal fungi might recruit some soil bacteria that could assist them in completing various ecological functions to form a specific endofungal bacterial microbiota in the fruiting body.

## MATERIALS AND METHODS

### Study sites and fruiting body sampling.

Fungal fruiting bodies of *T. neofelleus* were collected from Thousand Island Lake in July 2019 (29°34′45.67′′W, 118°54′45.71′′E). Only mature fruiting bodies were sampled, excluding immature and decaying samples. Mycosphere soil samples (~2 g) underneath and adhered to the fruiting body stalk were collected.

### Microorganisms, strains, and culture conditions.

Mycelia of *T. neofelleus* were isolated from the fruiting body and cultivated on modified Pachlewski medium (P20Th-; 1 g L^−1^ tartrate, 1 g L^−1^ KH_2_PO_4_, 0.5 g L^−1^ MgSO_4_, 10 g L^−1^ glucose, 1 mL L^−1^ diluted Kanieltra micronutrient solution [1:10], and 15 g L^−1^ agar, pH 5.5) at 25°C for 4 weeks as the fungal inoculum.

Three pieces of fungal inner tissue (5 mm^3^) were obtained using a sterile scalpel, avoiding contact with the outer surface of the fruiting body, and subsequently cultivated in 1.5-mL tubes containing liquid LB medium (1 mL) (31). The liquid medium was diluted (between 10^−1^ and 10^−4^) after 3 days of incubation and cultivated on the surface of the corresponding agar medium for 7 days. Single colonies were collected and purified based on their different morphologies from the agar medium. The 16S rRNA gene of each isolate was amplified using the universal bacterial primers 27F (5′-AGAGTTTGATCCTGGCTCAG-3′) and 1492R (5′-CTACGGCTACCTTGTTACGA-3′) under the following PCR conditions: 94°C for 5 min, 35 cycles of 94°C for 30 s, 50°C for 30 s, 72°C for 1 min, and a final elongation step at 72°C for 10 min (14). Seven strains were identified and stored in 50% glycerol at −80°C (Table S1 in the supplemental material).

### High-throughput sequencing of endofungal bacterial microbiota and mycosphere bacterial microbiota.

Total DNA was extracted from each mycosphere soil sample and fungal inner tissue with the commercial FastDNA spin kit for soil (MP Biomedicals, USA) according to the manufacturer’s protocols. Illumina MiSeq sequencing was performed for the detection of endofungal bacterial microbiota in fruiting bodies and mycosphere soil using an Illumina MiSeq PE300 platform at Majorbio Bio-pharm Technology Co., Ltd. (Shanghai, China). Primers 338F (5′-ACTCCTACGGGAGGCAGCAG-3′) and 806R (5′-GGACTACHGGGGTWTCTAAT-3′) were used to amplify the V3-V4 regions of the bacterial 16S rRNA gene (Gene Amp 9700, ABI, USA).

Sequences were quality filtered using Quantitative Insights into Microbial Ecology (QIIME) pipeline software (http://qiime.org/tutorials/tutorial.html), and low-quality reads were removed by Trimmomatic. Qualified sequences were clustered into operational taxonomic units (OTUs) at the 97% identity threshold using the Usearch program (version 7.1). The taxonomic classification of OTUs was annotated with the Ribosomal Database Project (RDP) classifier (http://rdp.cme.msu.edu/), and the obtained data were used for subsequent analyses. NMDS was implemented using the vegan package in the R version 3.20 environment (R Foundation for Statistical Computing, Vienna, Austria). The molecular functions of microbiota from each sample were predicted by PICRUSt based on KEGG databases. All raw reads were archived in the NCBI Sequence Read Archive database (accession number PRJNA882605).

### Dissolution mechanism of chelated inorganic phosphorus by the combined system.

Seven endofungal bacterial strains were cultured in liquid LB medium for 12 h at 200 rpm and 37°C and centrifuged at 5,000 × *g* for 10 min. To prepare a bacterial inoculum, cells were collected and washed twice in normal saline (0.85% NaCl) at 5,000 × *g* for 5 min and resuspended in saline to the final inoculum concentration. The dissolution ability of seven endofungal bacterial strains on chelated inorganic phosphorus was determined in the four treatments as follows: bacteria-only treatment, 10 μL of different bacterial inocula (10^6^ CFU mL^−1^) was added to 50 mL of modified National Botanical Research Institute’s phosphate (NBRIP) medium [5 g L^−1^ MgCl_2_·6H_2_O, 0.25 g L^−1^ MgSO_4_·H_2_O, 0.2 g L^−1^ KCl, 0.1 g L^−1^ (NH_4_)_2_SO_4_, 15 g L^−1^ agar, 10 g L^−1^ glucose, and 5 g L^−1^ Ca_3_(PO_4_)_2_, pH 7]; *T. neofelleus*-only treatment, 5-mm clumps of *T. neofelleus* inoculum were added to 50 mL of NBRIP medium; combined treatment, 10 μL of different bacteria inocula and 5-mm clumps of *T. neofelleus* inoculum were added to 50 mL of NBRIP medium; control treatment, 50 mL of NBRIP medium without bacteria and *T. neofelleus* inoculum. All treatments were incubated on a rotary shaker (180 rpm and 30°C).

Finally, we selected the endofungal bacteria *Bacillus* sp. strain B5 to further study the dissolution characteristics and mechanism of chelated inorganic phosphorus in the combined system of endofungal bacteria and *T. neofelleus* because it could adhere to the mycelial surface of *T. neofelleus* to establish a combined system, which had the highest soluble phosphorus content in the dissolution experiment of chelated inorganic phosphorus (Fig. S1 and S2). Samples of *Bacillus* sp. strain B5-only treatment, *T. neofelleus*-only treatment, and the combined treatment of *Bacillus* sp. strain B5 and *T. neofelleus* were taken every 2 days to determine the content of soluble phosphorus using the molybdenum blue method (32), organic acid, and the biomass of bacteria.

Organic acids in different treatments were analyzed by HPLC as described by Xu et al. (31). HPLC analysis was performed using an Agilent 1200 HPLC system with an Agilent HC-C_18_ column (4.6 × 250 mm, 5 μm; Agilent, USA) and ultraviolet absorption detector (UVD). The mobile phases were A (0.05 M H_2_SO_4_ [adjusted pH to 2.75 with H_2_SO_4_]) and B (methanol). HPLC conditions were as follows: 95% A and 5% B at a flow rate of 0.5 mL min^−1^ and detection at 210 nm with an oven temperature of 30°C. The solutions were filtered with a 0.22-μm filter before HPLC injection.

The activities of acid phosphatase (ACP), alkaline phosphatase (ALP), and phytase were determined in the culture after filtration through a 0.22-μm membrane (Merck Millipore) (33–35).

### Transcriptomic analysis by RNA-seq and RT-qPCR.

For RNA-seq, the combined treatment of *Bacillus* sp. strain B5 and *T. neofelleus* and *Bacillus* sp. strain B5-only treatment was sampled at 4 days according to the dissolution assay of chelated inorganic phosphorus. Harvested bacterial cells were obtained after removing the fungal clump (5,000 × *g* for 10 min), immediately frozen with liquid nitrogen, and stored at −80°C. The methods for RNA extraction, sequencing, and annotation are described in the Supplementary Appendix. The experiment was performed with three individual replicates. The RNA-seq reads were deposited in the Sequence Read Archive of the GenBank database under accession number PRJNA880718.

Total RNA from frozen *Bacillus* sp. strain B5 cells was extracted using an RNeasy mini kit (Qiagen) according to the manufacturer’s instructions. To eliminate genomic DNA contamination, the extracted RNA was treated with DNase at 42°C for 60 s. The integrity and concentration of RNA were determined by gel electrophoresis and a NanoDrop 2000 spectrophotometer (Thermo), respectively. Approximately 500 ng of RNA was used to generate the first-strand cDNA using a HiScript II first strand cDNA synthesis kit (Vazyme Biotech Co., Ltd.). RT-qPCR was performed on a 7500 real-time PCR system (Applied Biosystems) with AceQ qPCR SYBR green master mix (Vazyme Biotech Co., Ltd.). The PCR conditions were as follows: 95°C for 5 min and 40 cycles of 95°C for 10 s, 60°C for 30 s, and 72°C for 20 s. A melting curve was recorded at the end of each run to verify the specificity of the reaction. The 16S rRNA gene of *Bacillus* sp. strain B5 was used as a reference gene to normalize the expression of selected genes using the cycling threshold (2^−△△^*^CT^*) method.

### Pot experimental design.

The microcosm was composed of two main compartments (8 × 8.7 × 11.4 cm for the plant compartment and 3 × 8.7 × 11.4 cm for the hyphal compartment) separated by 30-μm nylon mesh and a 1-cm buffer compartment from the mesh toward the hyphal compartment (Fig. S3). There were 340 g, 40 g, and 130 g of vermiculite in the plant compartment, buffer compartment, and hyphal compartment with 2.5 μg g^−1^ Ca_3_(PO_4_)_2_, respectively. *Pinus sylvestris* seeds were surface sterilized with 70% (vol/vol) ethanol for 3 min and 3% (vol/vol) sodium hypochlorite for 3 min and washed three times with sterilized distilled water (repeated three times). The surface-sterilized seeds germinated in the dark at 28°C on sterilized vermiculite, and five seedlings were transplanted to the root compartment after being cultivated for 28 days. Furthermore, *T. neofelleus* inoculum (5 g) was added into the plant compartment when needed in different treatments, while the autoclaved *T. neofelleus* inoculum (5 g) served as a control. The *Bacillus* sp. strain B5 inoculum (10^9^ CFU mL^−1^; 5 mL) was added into the hyphal compartment when needed in different treatments, while the autoclaved *Bacillus* sp. strain B5 inoculum served as a control. Thus, there were four treatments as follows: control treatment, no inoculation of *T. neofelleus* and *Bacillus* sp. strain B5; *T. neofelleus*-only treatment, inoculation with *T. neofelleus* and sterilized *Bacillus* sp. strain B5; *Bacillus* sp. strain B5-only treatment, inoculation with *Bacillus* sp. strain B5 and sterilized *T. neofelleus*; combined treatment, inoculation with *T. neofelleus* and *Bacillus* sp. strain B5. All treatments were cultivated in a controlled environment greenhouse (28°C, 16-h/8-h light/dark cycle, with light at 450 μmol m^−2^ s^−1^) at Nanjing Normal University (Nanjing, China, 32°11′N, 118°91′E) for 90 days. Fresh sterilized phosphorus-free Hoagland nutrient solution [607 mg K_2_SO_4_, 493 mg Mg_2_SO_4_, 66.02 mg (NH_4_)_2_SO_4_, 20 mg C_10_H_12_FeN_2_NaO_8_, 15 mg FeSO_4_, 2.86 mg H_3_BO_3_, 4.5 mg Na_2_B_4_O_7_·10H_2_O, 2.13 mg MnSO_4_, 0.05 mg CuSO_4_, 0.22 mg ZnSO_4_, and 945 mg Ca(NO_3_)_2_] was added every 2 weeks. The ectomycorrhizal structure was observed using the toluidine blue method after 3 months (36).

### Plant growth parameters and vermiculite properties.

Plant growth parameters, including root length, shoot length, dry weight, and the number of lateral roots, were measured at 90 days after planting. The dried plant sample (0.1 g) was ground and digested with H_2_SO_4_-H_2_O_2_ solution. Inorganic phosphorus concentrations in the digestions and extracts were determined using the molybdenum blue method. Moreover, the lateral roots of *P. sylvestris* seedlings randomly selected from each treatment were embedded in paraffin, and the cross sections were taken for the determination of the ectomycorrhizal colonization rate based on the presence of mantles or Hartig net structures.

The vermiculite from the three compartments was collected and air dried. The dried vermiculite (0.2 g) was digested with H_2_SO_4_-H_2_O_2_ solution and extracted with 0.5 mol L^−1^ NaHCO_3_ for Olsen P. Inorganic phosphorus concentrations in the digestions and extracts were determined using the molybdenum blue method (32). Soil pH was determined with a pH meter (PHS-3C; Hangzhou Qiwei Instrument Co., Ltd., China) with a water-to-soil ratio of 2.5:1 (vol:wt).

### Statistical analyses.

The data are expressed as mean ± standard error (SE). Statistical analyses were performed with SPSS v. 18.0 (SPSS, Inc., Chicago, IL, USA) using Kruskal-Wallis analysis of variance, *post hoc* Tukey and least significant difference (LSD) multiple range test, Mann-Whitney *U* test, independent sample *t* test, and one-way analysis of variance via Duncan’s honestly significant difference, as appropriate.

### Data availability.

All raw reads of high-throughput sequencing were archived in the NCBI Sequence Read Archive database (accession number PRJNA882605). The RNA-seq reads were deposited in the Sequence Read Archive of the GenBank database (accession number PRJNA880718).
