# Supplementary material for: Endofungal Bacterial Microbiota Promotes the Absorption of Chelated Inorganic Phosphorus by Host Pine through the Ectomycorrhizal System
Source: Microbiol Spectr. 2023 Jul 5;11(4):e00162-23. doi: 10.1128/spectrum.00162-23 (PMC10433794; doi:10.1128/spectrum.00162-23)
Supplement: Supplemental file 1 — Supplemental material. Download spectrum.00162-23-s0001.docx, DOCX file, 13.1 MB [file spectrum.00162-23-s0001.docx]

**Supplementary Information for :** Endofungal bacterial microbiota promotes the absorption of chelated inorganic phosphorus by host pine through the ectomycorrhizal system

Aiyue Zhang^a^, Meiling Zhang^a^, Jiale Zhu^a^, Yan Mei^a^, Fangji Xu^b^, Hongyan Bai^a^, Kai Sun^a^, Wei Zhang^a^, Chuanchao Dai^a^, Yong Jia^a,*^

**This file includes:**

Supplementary Table S1-S3

Supplementary Figure S1-S16

Supplementary Appendix

Supplementary table S1. The information of seven endofungal bacterial strains [1]

| Identification by 16s rRNA gene | Accession |
| --- | --- |
| *Pseudomonas* sp. strain B25 | MZ425420.1 |
| *Pseudomonas aeruginosa* strain B24 | MZ425419.1 |
| *Bacillus altitudinis* strain B14 | MZ425415.1 |
| *Bacillus* sp.strain B1 | MZ425402.1 |
| *Bacillus aerius* strain B3 | MZ425404.1 |
| *Bacillus* sp. strain B5 | MZ425406.1 |
| *Stenotrophomonas* sp. strain B31 | MZ425425.1 |

**References**

1. Bai H-Y, Zhang A-Y, Mei Y, Xu M, Lu X-L, Dai C-C, et al. Effects of ectomycorrhizal fungus bolete identity on the community assemblages of endofungal bacteria. ENVIRONMENTAL MICROBIOLOGY REPORTS. 2021;13(6):852-61.

Supplementary table S2. Alkaline phosphatase activity, Phytase activity, and Acid phosphatase activity of *Bacillus* sp. strain B5

| Treatment | AKP activity （U） | | | Phytase activity (U) | | | ACP activity (U) | | |
| --- | --- | --- | --- | --- | --- | --- | --- | --- | --- |
|  | B | F | BF | B | F | BF | B | F | BF |
| 0d | 0.16±0.09a | 0.14±0.13a | 0.17±0.01a | - | 0.07±0.02a | 0.09±0.00a | - | 0.34±0.11a | 0.33±0.03a |
| 2d | 0.19±0.06a | 0.18±0.06a | 0.26±0.05a | - | 0.12±0.01a | 0.13±0.02a | - | 0.37±0.03a | 0.36±0.07a |
| 4d | 0.2±0.02a | 0.19±0.08a | 0.21±0.06a | - | 0.15±0.01a | 0.16±0.01a | - | 0.40±0.02a | 0.41±0.01a |
| 6d | 0.22±0.02a | 0.20±0.01a | 0.21±0.09a | - | 0.22±0.06a | 0.26±0.10a | - | 0.42±0.03a | 0.43±0.10a |
| 8d | 0.21±0.03a | 0.20±0.08a | 0.23±0.09a | - | 0.23±0.00a | 0.25±0.03a | - | 0.52±0.03a | 0.49±0.05a |
| 10d | 0.21±0.03a | 0.24±0.01a | 0.25±0.04a | - | 0.25±0.01a | 0.24±0.03a | - | 0.50±0.07a | 0.53±0.07a |

B, *Bacillus* sp. strain B5-only treatment; F, *T. neofelleus*-only treatment; BF, the combined treatment of *Bacillus* sp. strain B5 and *T. neofelleus*. Values were the mean of three biological replicates ± SE. The different letter showed that there was significant difference between the groups as analyzed by one-way ANOVA, Tukey’s multiple range test and Kruskal-Wallis analysis of variance, post-hoc Dunn-Bonferroni’s multiple range test (*p* < 0.05).“-”indicated no related enzyme activity in *Bacillus* sp. strain B5-only treatment.

Supplementary table S3. Ectomycorrhizal colonization rate (%) in the *Pinus sylvestris* seedlings colonized by *T. neofelleus*

|  | CK | B5 | F | B5F |
| --- | --- | --- | --- | --- |
| Ectomycorrhizal colonization rate | 0 | 0 | 71.43%±14.29%a | 80.95%±8.25%a |

B, *Bacillus* sp. strain B5-only treatment; F, *T. neofelleus*-only treatment; BF, the combined treatment of *Bacillus* sp. strain B5 and *T. neofelleus*. Values were the mean of three biological replicates ± SE. The same letter showed that there was no significant difference between the groups as analyzed by Independent sample t-test (*p* =0.561).


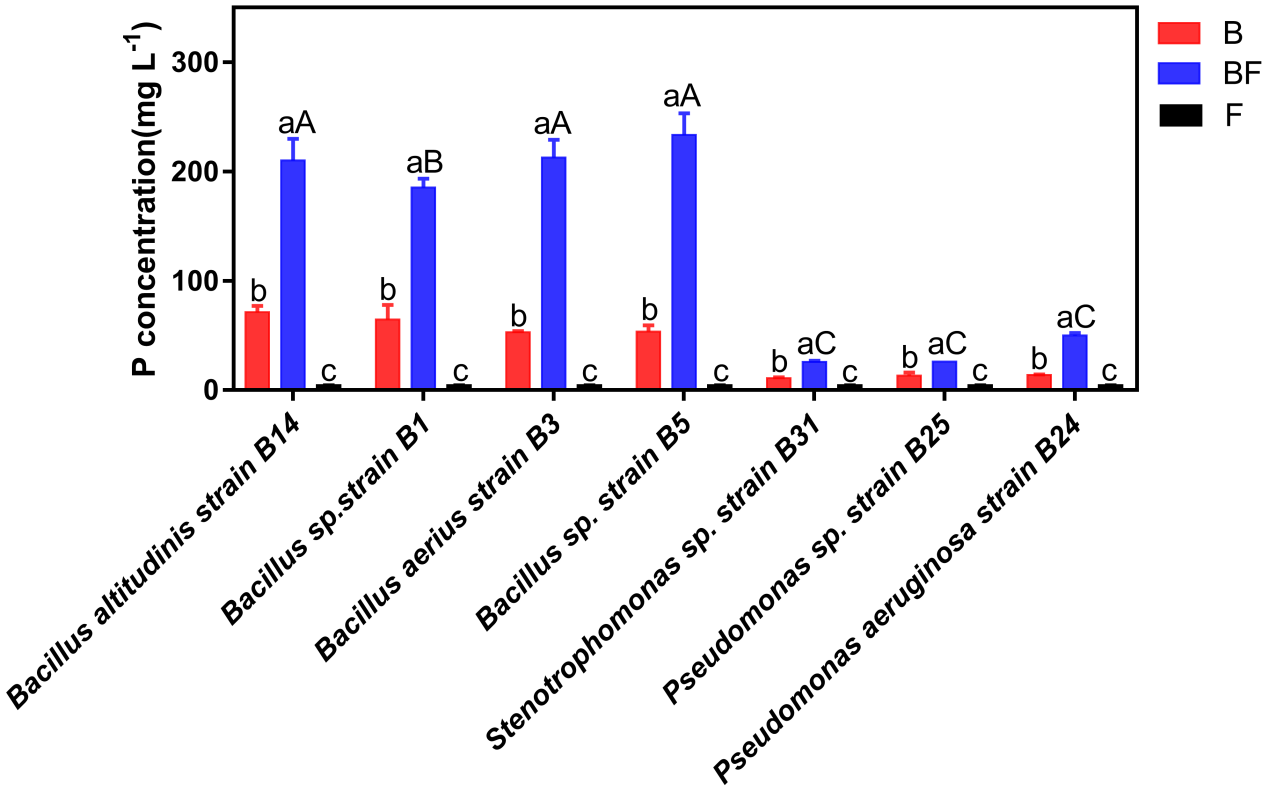


Supplementary Fig. S1 The chelated inorganic phosphorus dissolution ability of cultivable endofungal bacteria and *T. neofelleus*. Values were the mean of three biological replicates ± SE. The different lowercase letter on the top of the column showed that there was significant difference among the three different treatments as analyzed by Kruskal-Wallis analysis of variance, post-hoc Dunn-Bonferroni’s multiple range test, *p* < 0.05. The different capital letter on the top of the column showed that there was significant difference among the combined treatment of differernt bacterial strain as analyzed by Kruskal-Wallis analysis of variance, post-hoc Tukey and LSD’s multiple range test, *p* < 0.05. B, bacteria-only treatment; F, only *T. neofelleus* treantment; BF, the combined treatment of bacteria and *T. neofelleus* treatment.


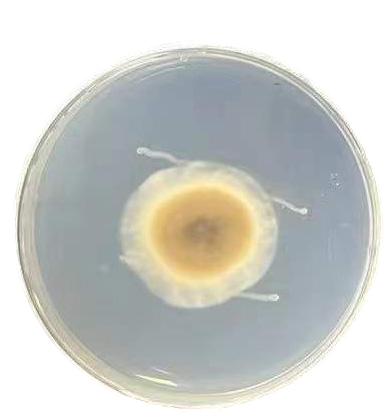

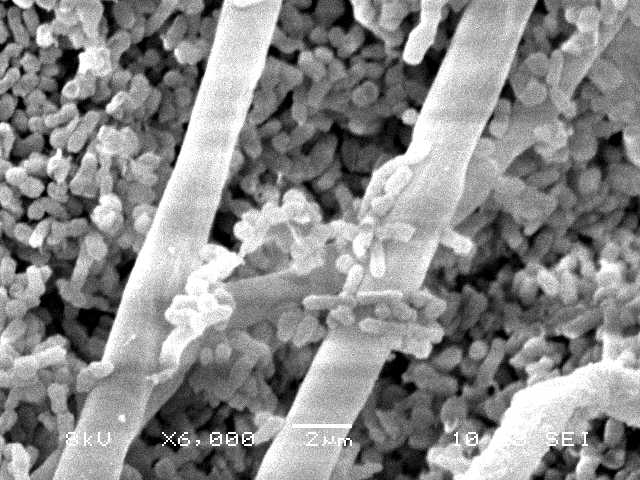


Supplementary Fig. S2 Co-culture plate diagram of *Bacillus* sp. strain B5 and *T. neofelleus* and the contact sites by scanning electron microscopy.


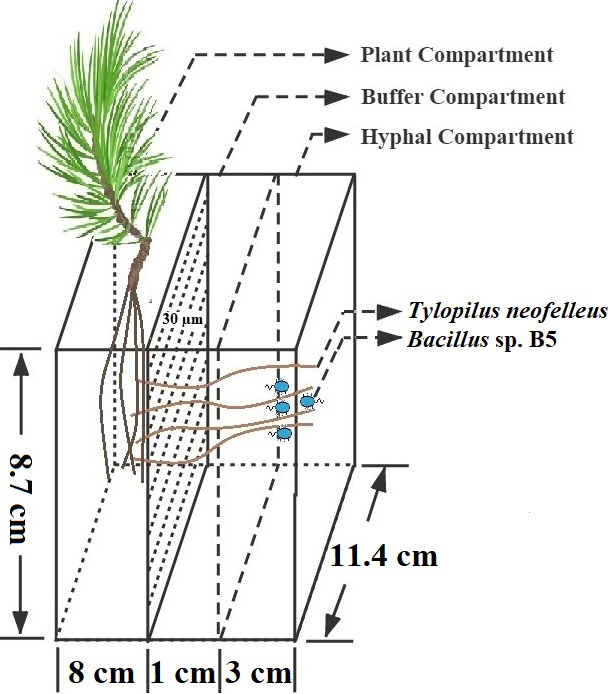


Supplementary Fig. S3 The microcosm of pot experiment, including plant compartment, buffer compartment, and hyphal compartment.


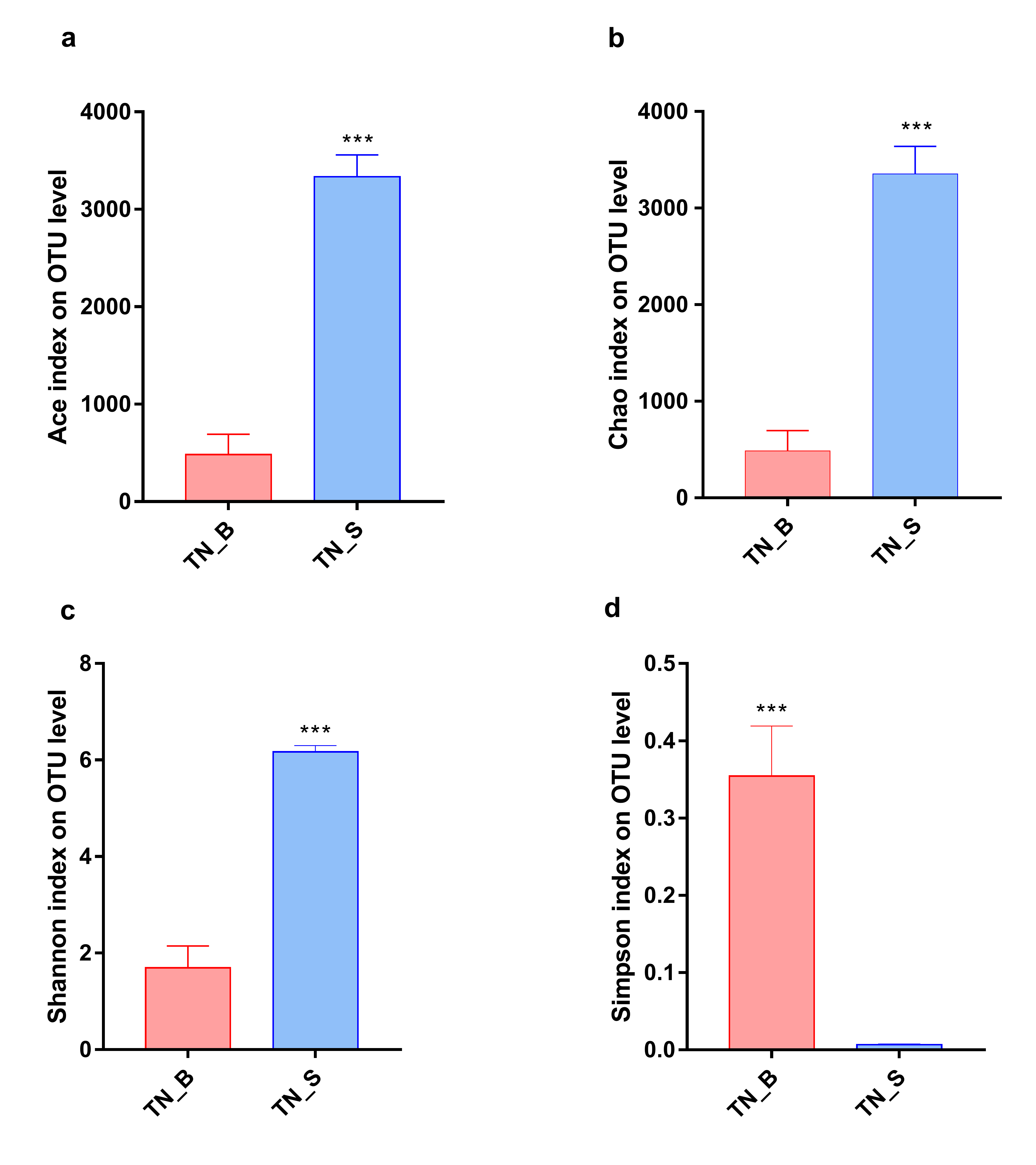


Supplementary Fig. S4 Bacterial α diversity of the endofungal bacterial microbiota in *T. neofelleus* fruiting body and the corresponding mycosphere soil bacterial microbiota at OTU level. a, ACE index; b, Chao index; c, Shannon index; d, Simpson index. Values were the mean of three biological replicates ± SE. The different letter on the top of the column showed that there was significant difference between the groups as analyzed by Independent sample t-test (a, b, c) and Mann-Whitney U test (d) (**p* < 0.05，***p* < 0.01，*** *p* < 0.001). TN_B = Endofungal bacterial microbiota of *Tylopilus neofelleus*; TN_S = *Tylopilus neofelleus* mycosphere soil bacterial microbiota.


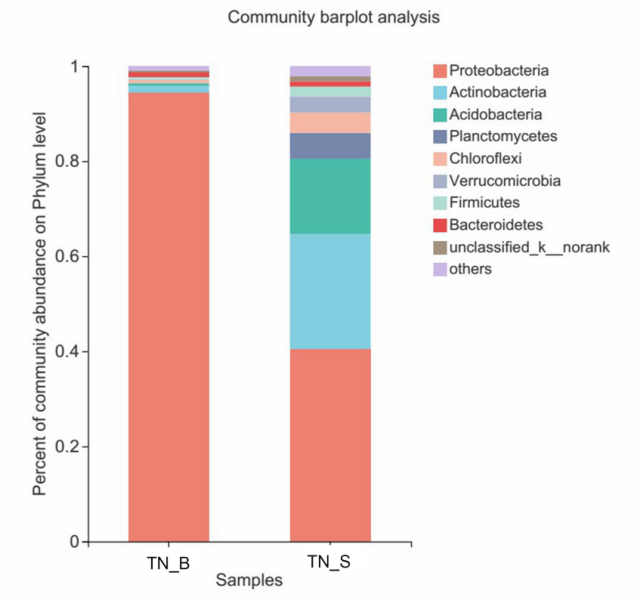

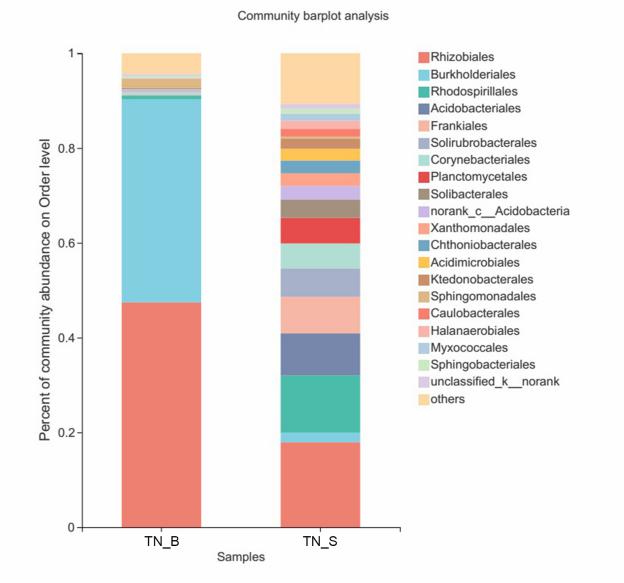

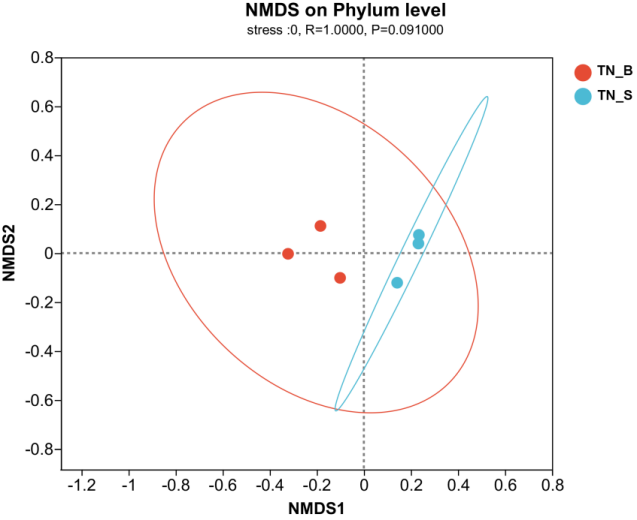

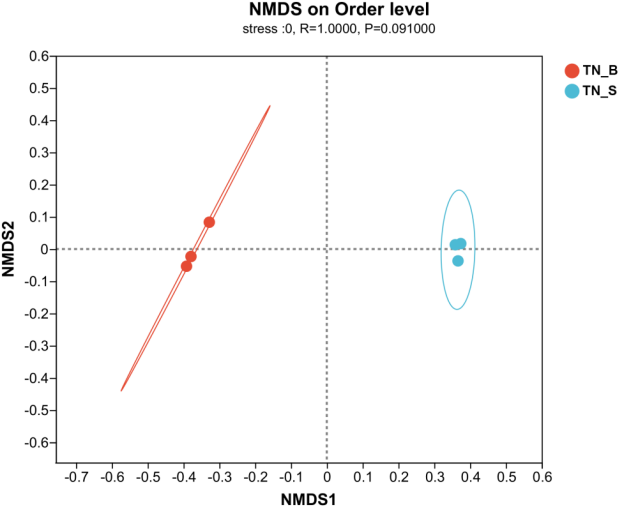


d

c

b

a

Supplementary Fig. S5 The structure of the endofungal bacterial microbiota in *T. neofelleus* fruiting body and the corresponding mycosphere soil bacterial microbiota. a, The relative abundance of endofungal bacterial microbiota and mycosphere soil bacterial microbiota at phylum level; b, The relative abundance of endofungal bacterial microbiota and mycosphere soil bacterial microbiota at order level; c, Nonmetric multidimensional scaling analyses (NMDS) of Bray-Curtis distance of bacterial microbiota in fruiting body and mycosphere soil at the phylum level; d, Nonmetric multidimensional scaling analyses (NMDS) of Bray-Curtis distance of bacterial microbiota in fruiting body and mycosphere soil at the order level. TN_B = Endofungal bacterial microbiota of *Tylopilus neofelleus*; TN_S = *Tylopilus neofelleus* mycosphere soil bacterial microbiota.


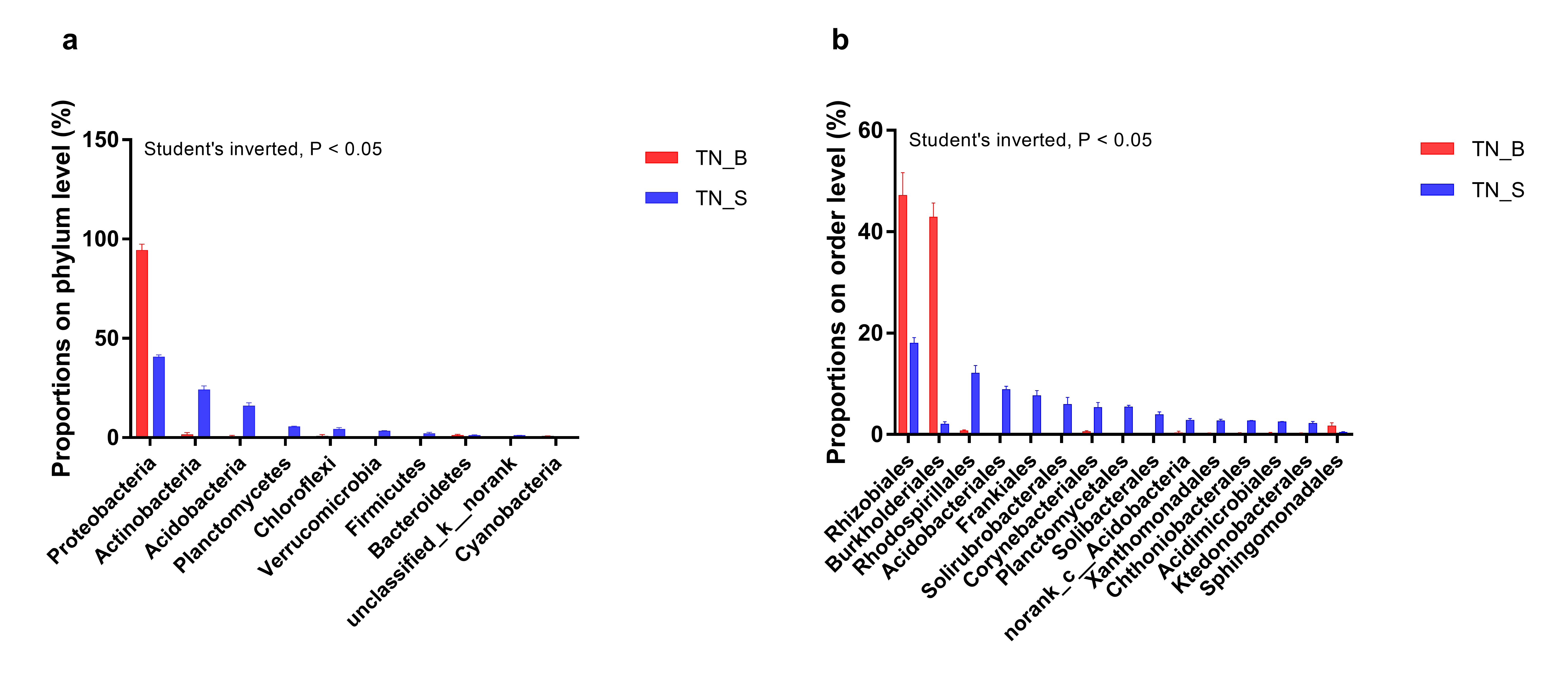


Supplementary Fig. S6 Differential abundance between the endofungal bacterial microbiota in *T. neofelleus* fruiting body and the corresponding mycosphere soil bacterial microbiota at phyla and order. Student’s T test followed by FDR (*p* < 0.05) corrections were performed at phylum (a) and order level (b). TN_B = Endofungal bacterial microbiota of *Tylopilus neofelleus*; TN_S = *Tylopilus neofelleus* mycosphere soil bacterial microbiota.


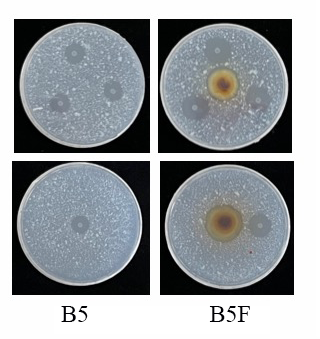


Supplementary Fig. S7 Plate experiments on the solubilization of chelated inorganic phosphorus state by the *Bacillus* sp. strain B5-only treatment and the combined treatment of *Bacillus* sp. strain B5 and *T. neofelleus*. B, *Bacillus* sp. strain B5-only treatment; B5F, the combined treatment of *Bacillus* sp. strain B5 and *T. neofelleus*.


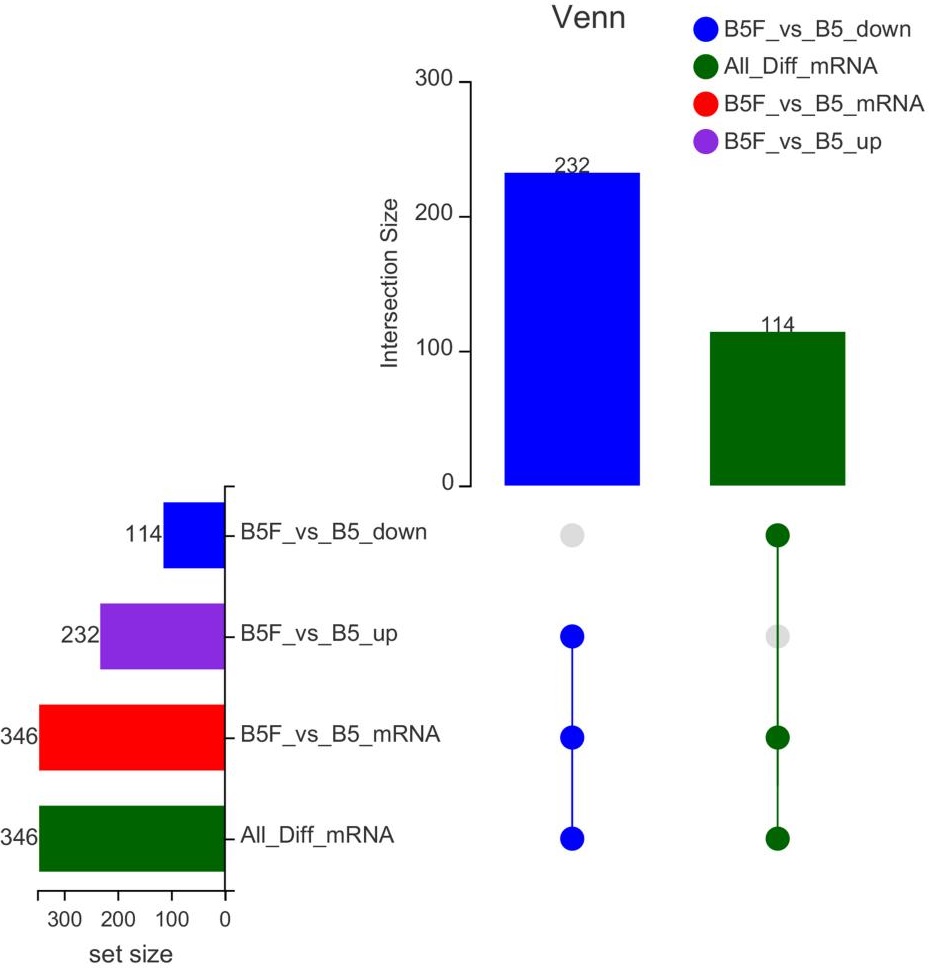


Supplementary Fig. S8 Transcriptomic analysis between the only *Bacillus* sp. strain B5 treatment and the combined treatment of *Bacillus* sp. strain B5 and *T. neofelleus*. B, *Bacillus* sp. strain B5-only treatment; B5F, the combined treatment of *Bacillus* sp. strain B5 and *T. neofelleus*.

**
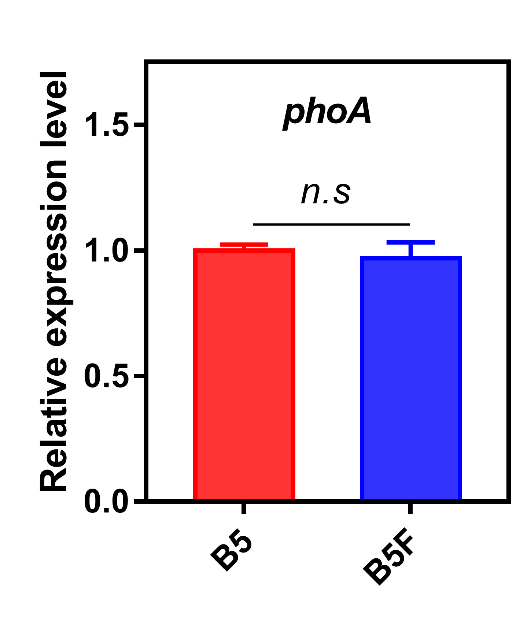
**

Supplementary Fig. S9 Alkaline phosphatase gene expression of *Bacillus* sp. strain B5. Values were the mean of three biological replicates ± SE (Mann-Whitney U test; *p* = 0.258 )


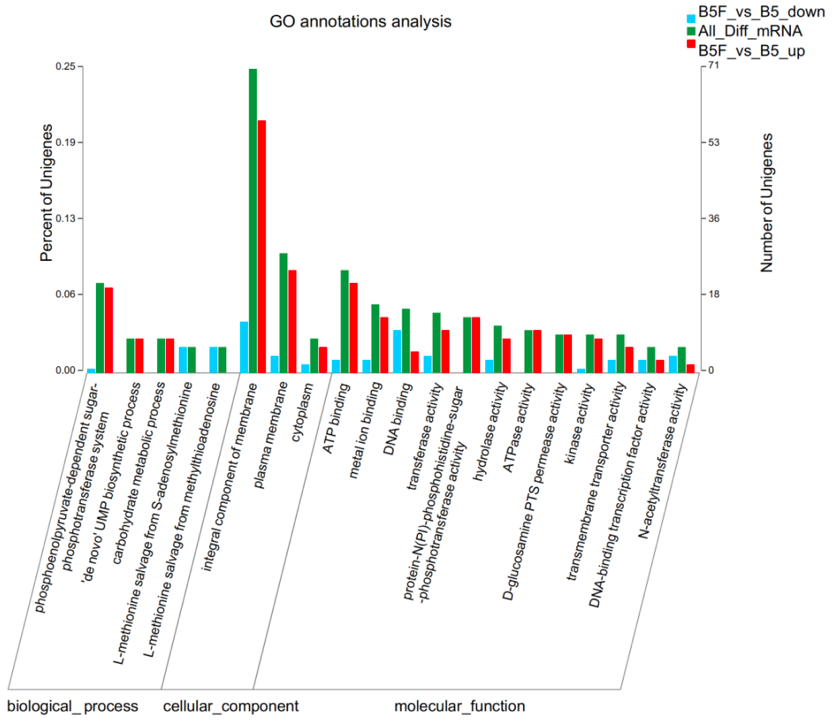


a

b


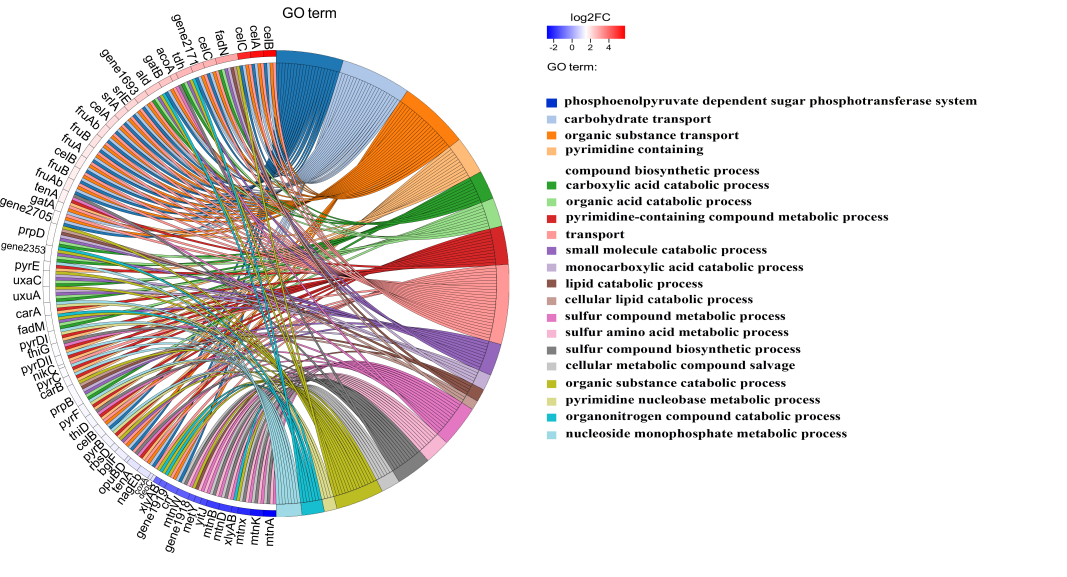


Supplementary Fig. S10 Gene Ontology (GO) analysis of differentially expressed genes of *Bacillus* sp. strain B5 between the *Bacillus* sp. strain B5-only treatment and the combined treatment of *Bacillus* sp. strain B5 and *T. neofelleus*. B, *Bacillus* sp. strain B5-only treatment; B5F, the combined treatment of *Bacillus* sp. strain B5 and *T. neofelleus*. a, The differentially expressed genes; b, Functional enrichment analysis of the first 20 gene sets.


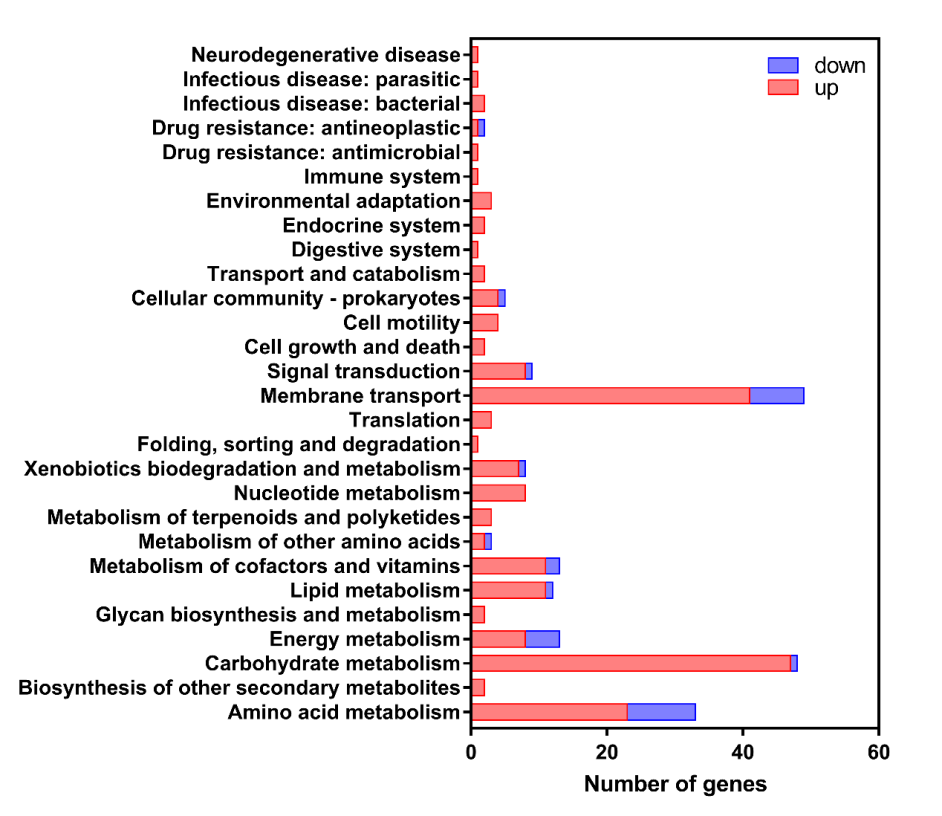


Supplementary Fig. S11 KEGG analysis of differentially expressed genes of *Bacillus* sp. strain B5 between the *Bacillus* sp. strain B5-only treatment and the combined treatment of *Bacillus* sp. strain B5 and *T. neofelleus* (B5F vs B5). B, *Bacillus* sp. strain B5-only treatment; B5F, the combined treatment of *Bacillus* sp. strain B5 and *T. neofelleus*.


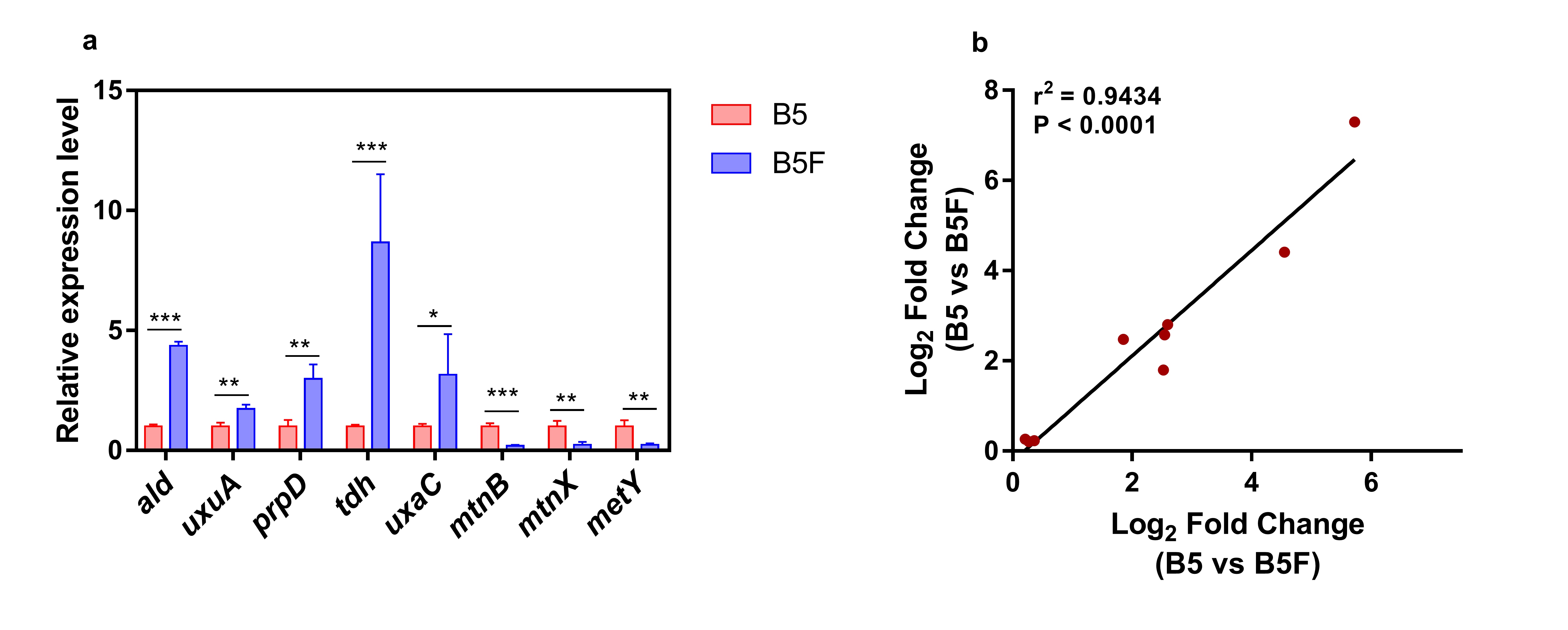


Supplementary Fig. S12 Validation of RNA-Seq by qPCR (a) and correlation of results from qRT-PCR (b). Values were the mean of three biological replicates ± SE. The different letter on the top of the column showed that there was significant difference between the groups as analyzed by Independent sample t-test (*ald, uxuA*, *prpD*, *mtnX*, *metY, uxaC*) and Mann-Whitney U test (*tdh*, *mtnB*). (**p* < 0.05，***p* < 0.01，*** *p* < 0.001). B, *Bacillus* sp. strain B5-only treatment; B5F, the combined treatment of *Bacillus* sp. strain B5 and *T. neofelleus*.


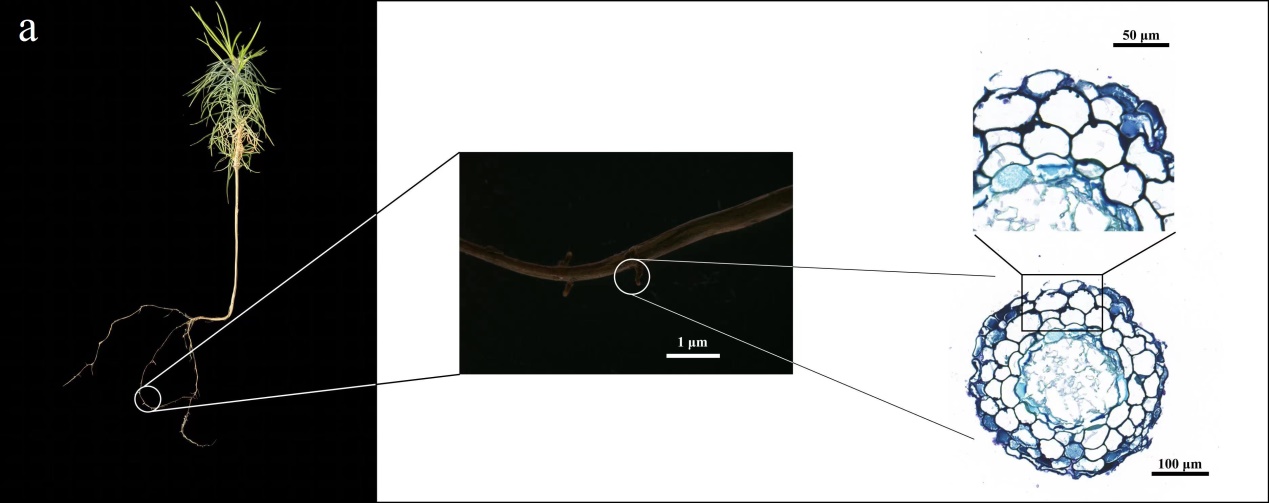


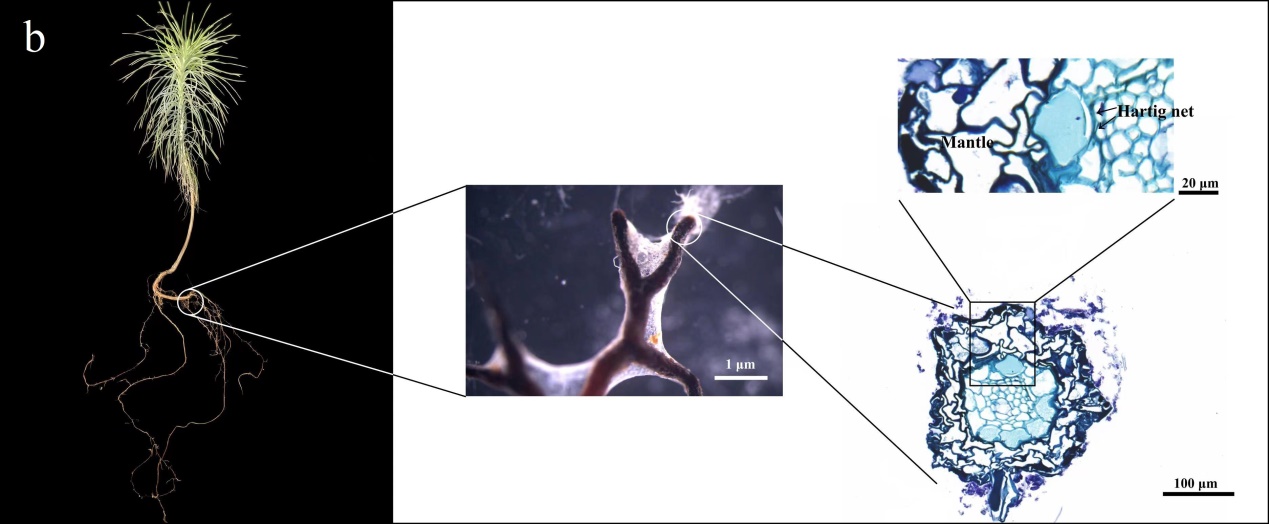


Supplementary Fig. S13 Root characterization and ectomycorrhizal structure of *Pinus sylvestris* under the *Bacillus* sp. strain B5-only treatment and the combined treatment of *Bacillus* sp. strain B5 and *T. neofelleus*. a, the root of *Pinus sylvestris* under the *Bacillus* sp. strain B5-only treatment; b, the root of *Pinus sylvestris* under the combined treatment of *Bacillus* sp. strain B5 and *T. neofelleus*.

a b


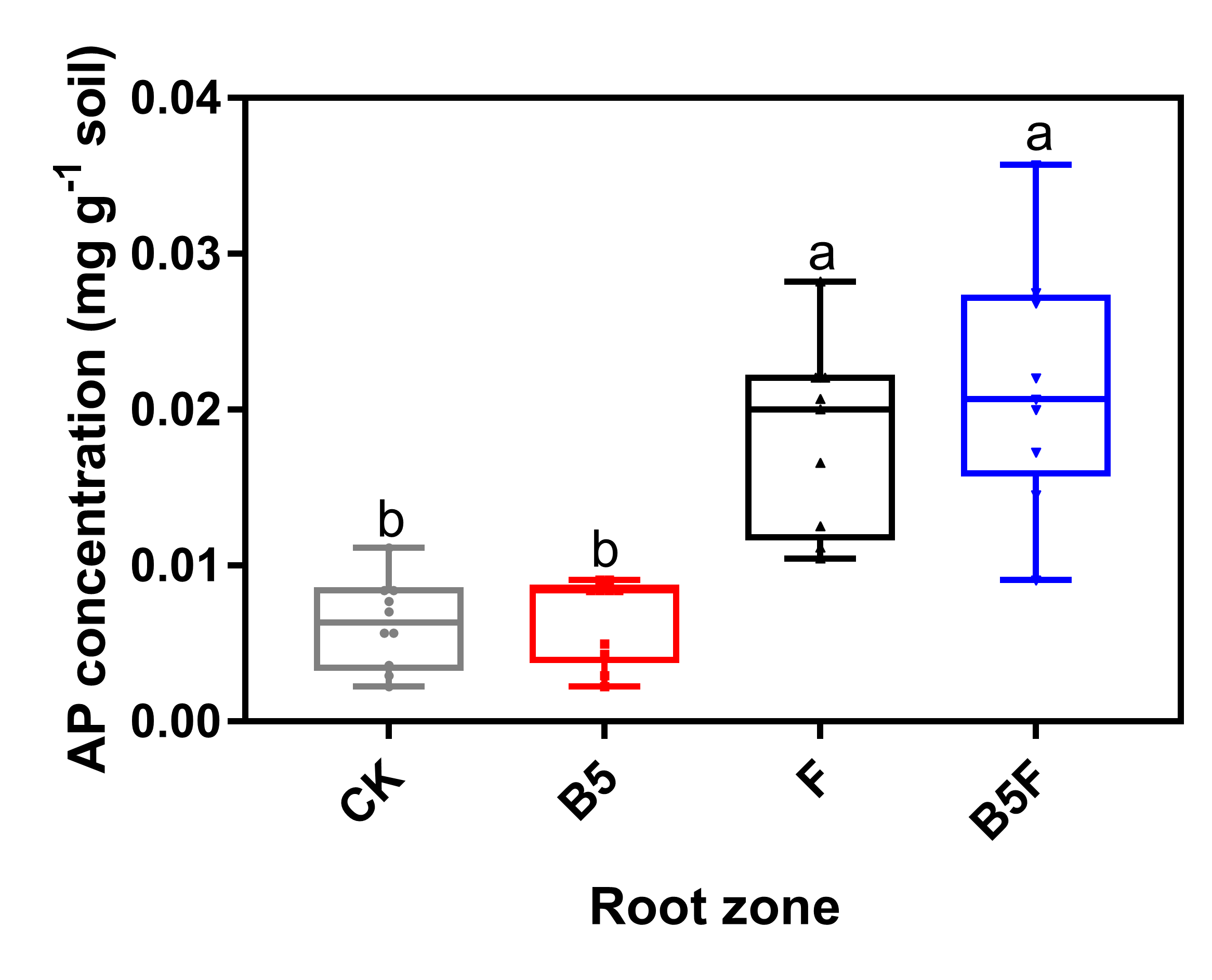

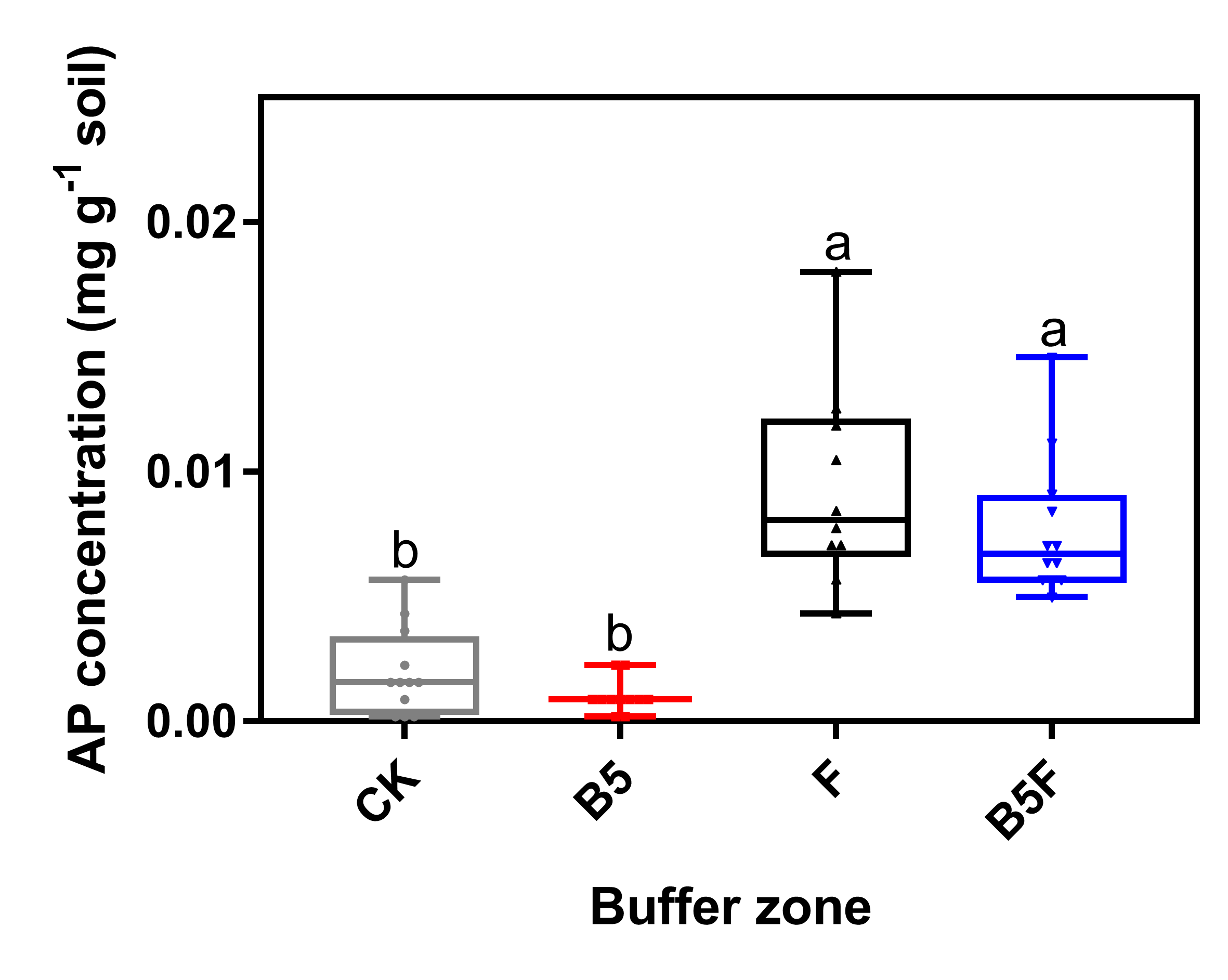


c d


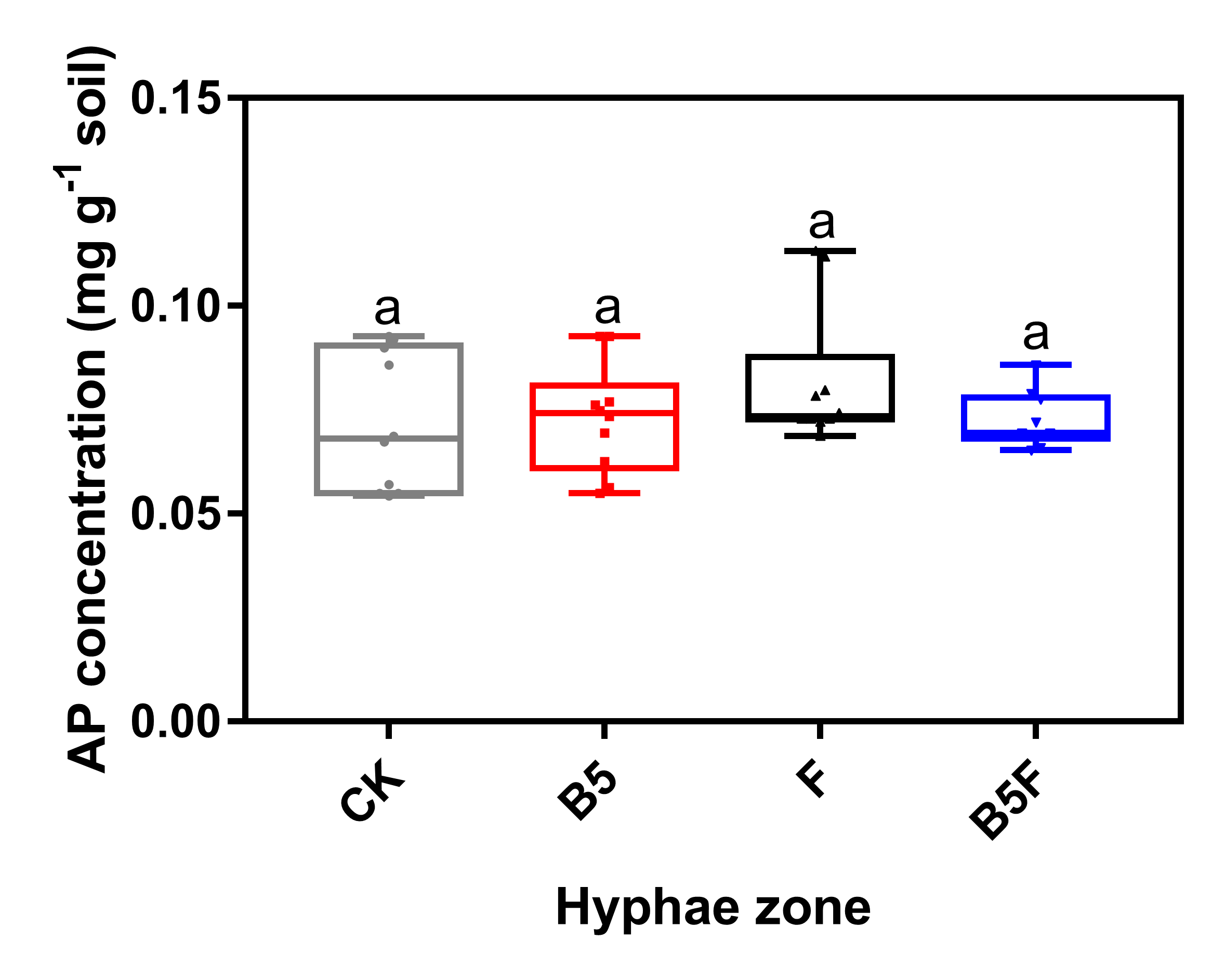

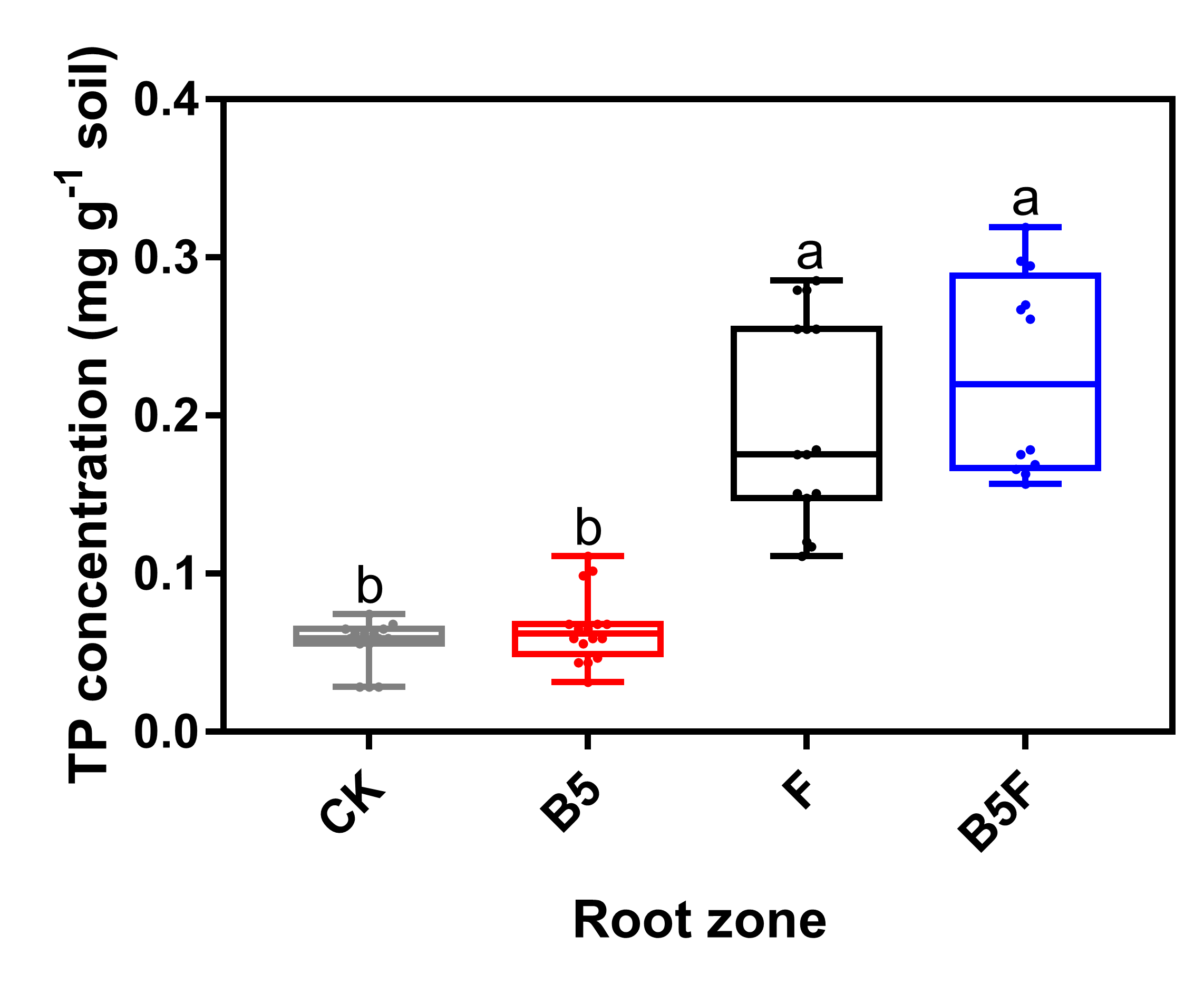


e f


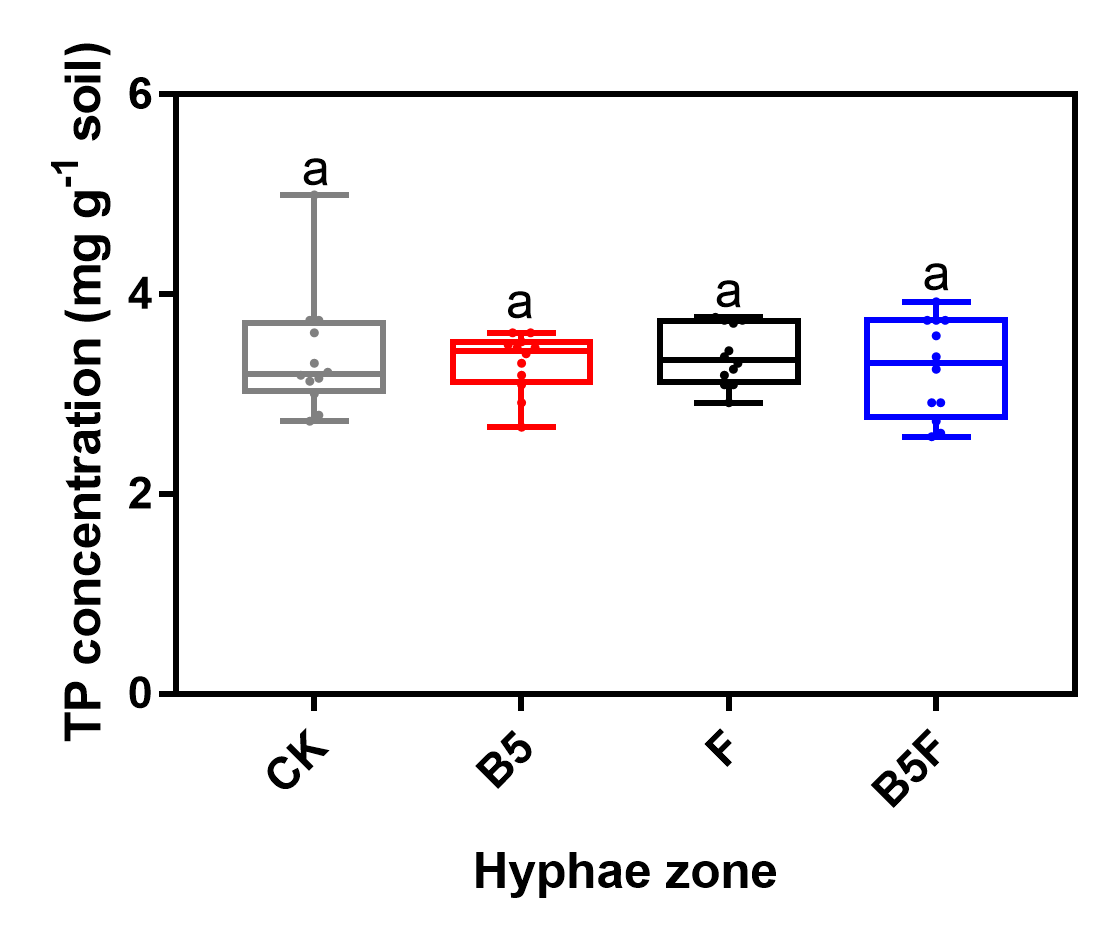

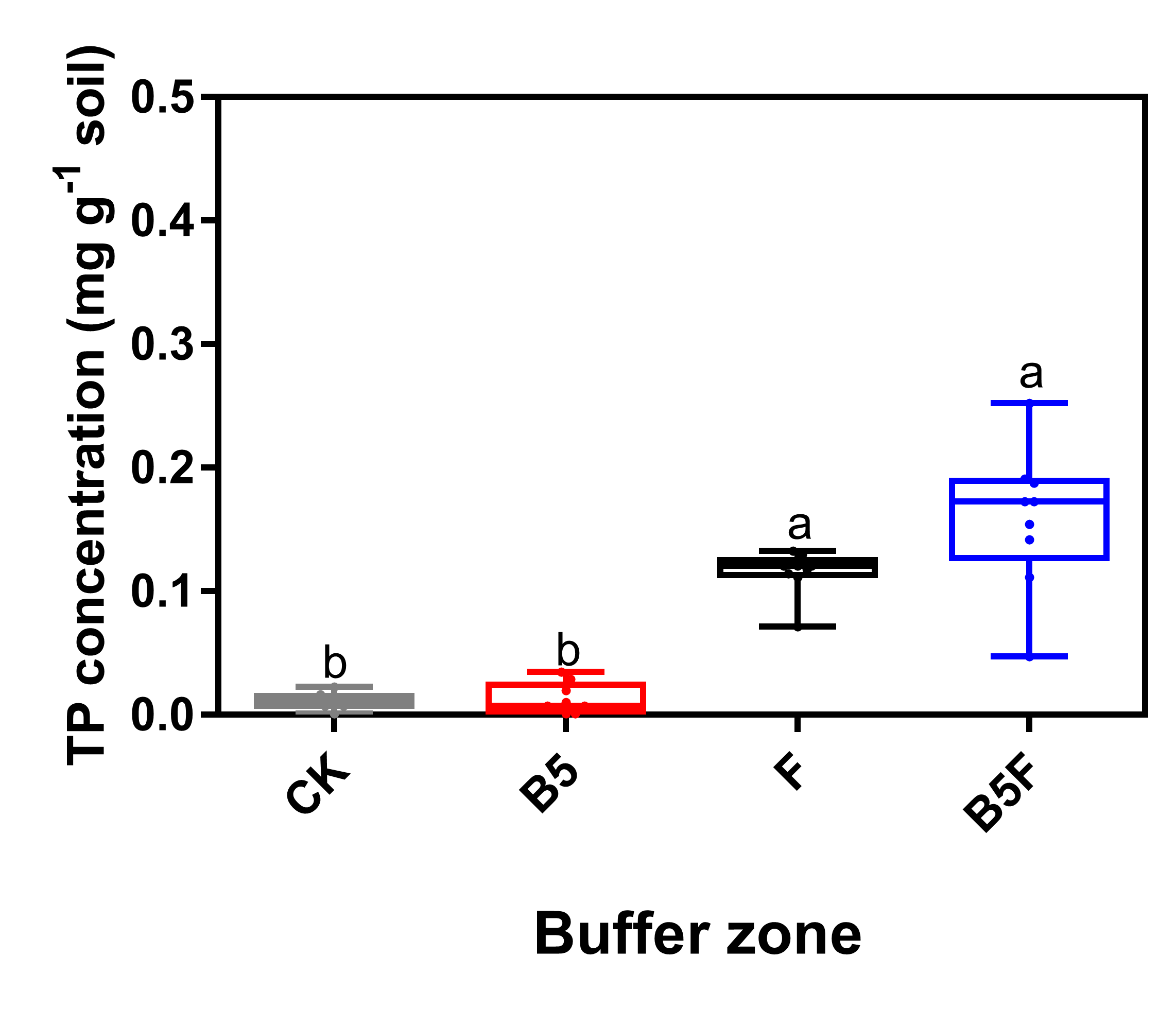


Supplementary Fig. S14 Determination of phosphorus content in different compartments. a, Available phosphorous concentrations of plant compartment; b, Available phosphorous concentrations of buffer compartment; c, Available phosphorous concentrations of hyphal compartment; d, Total phosphorous concentrations of plant compartment; e, Total phosphorous concentrations of buffer compartment; f, Total phosphorous concentrations of hyphal compartment. Values were the means ± SE of six independent replicates. Bar with different letters indicated statistically significant differences (Kruskal-Wallis analysis of variance, post-hoc Dunn-Bonferroni’s multiple range test, *p* < 0.05). CK, control treatment; B, *Bacillus* sp. strain B5-only treatment; F, *T. neofelleus*-only treatment; B5F, the combined treatment of *Bacillus* sp. strain B5 and *T. neofelleus*.


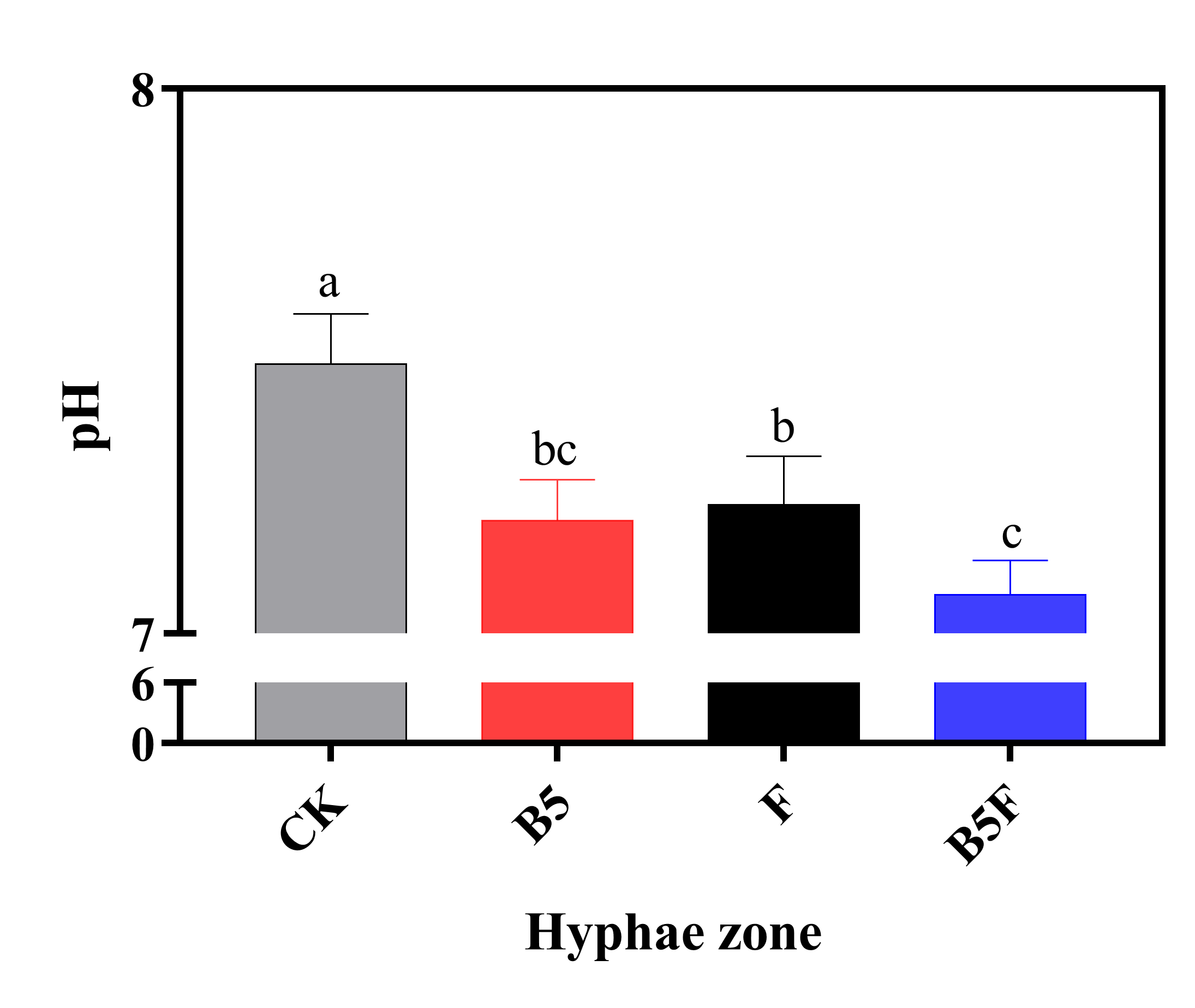


Supplementary Fig. S15 Determination of pH in different treatment groups in the hyphae zone. Values were the means ± SE of six independent replicates. Bar with different letters indicated statistically significant differences (one-way ANOVA, Duncan’s multiple range test, *p* < 0.05). CK, control treatment; B, *Bacillus* sp. strain B5-only treatment; F, *T. neofelleus*-only treatment; B5F, the combined treatment of *Bacillus* sp. strain B5 and *T. neofelleus*.


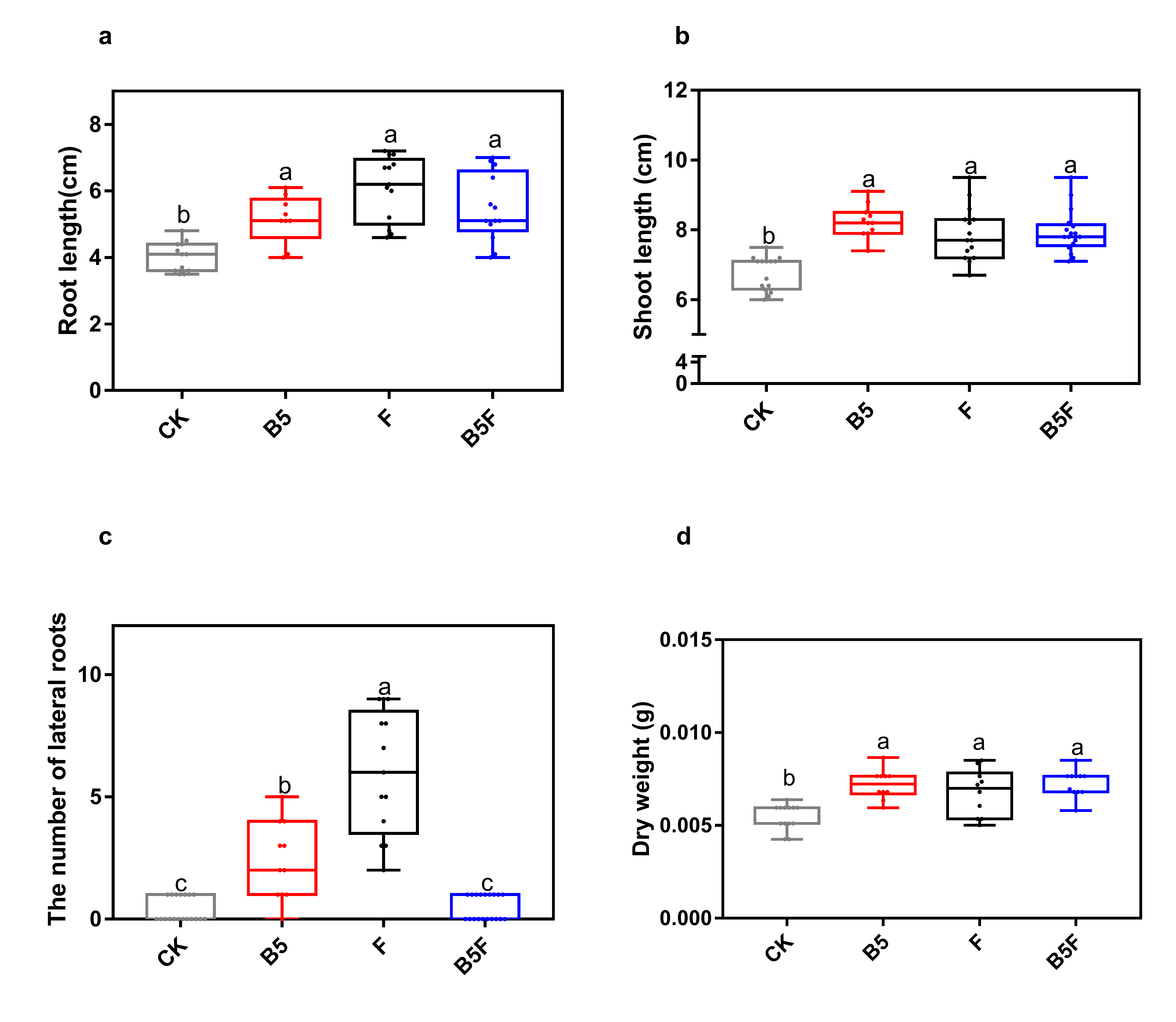


Supplementary Fig. S16 The growth properties of *Pinus sylvestris* seedling. a, Root length; b, Shoot length; c, The number of lateral roots; d, Dry weight. Values were the means ± SE of six independent replicates. Bar with different letters indicated statistically significant differences (a, b: one-way ANOVA, Duncan’s multiple range test, *p* < 0.05; c, d: Kruskal-Wallis analysis of variance, post-hoc Dunn-Bonferroni’s multiple range test, *p* < 0.05 ). CK, control treatment; B, *Bacillus* sp. strain B5-only treatment; F, *T. neofelleus*-only treatment; B5F, the combined treatment of *Bacillus* sp. strain B5 and *T. neofelleus*.

**Supplementary Appendix**

For RNA-seq, the *Bacillus* sp. strain B5 cells were collected in combined treatment and *Bacillus* sp. strain B5-only treatment at 4 days after inoculation. Harvested cells were immediately frozen with liquid nitrogen and stored at -80 ^o^C.

Total RNA was extracted from the tissue using TRIzol® Reagent according to the manufacturer’s instructions (Invitrogen), and genomic DNA was removed using DNase I (TaKara). Then RNA quality was determined by 2100 Bioanalyser (Agilent) and quantified using the ND-2000 (NanoDrop Technologies). Only high-quality RNA sample (OD260/280=1.8~2.0, OD260/230≥2.0, RIN≥6.5, 28S:18S≥1.0, ≥100ng/μl, ≥2μg) was used to construct the sequencing library.

RNA-seq transcriptome library was prepared following TruSeqTM RNA sample preparation Kit from Illumina (San Diego, CA) using 2μg of total RNA. Shortly, ribosomal RNA (rRNA) depletion instead of poly (A) purification is performed by Ribo-Zero Magnetic kit (epicenter), and then all mRNAs were broken into short (200nt) fragments by adding fragmentation buffer firstly. Secondly, double-stranded cDNA was synthesized using a SuperScript double-stranded cDNA synthesis kit (Invitrogen, CA) with random hexamer primers (Illumina). When the second strand cDNA was synthesized, dUTP was incorporated in place of dTTP. Then the synthesized cDNA was subjected to end-repair, phosphorylation, and ‘A’ base addition according to Illumina’s library construction protocol. The second strand cDNA with dUTP was recognized and degraded by UNG enzyme. Libraries were selected for cDNA target fragments of 200bp on 2% Low Range Ultra Agarose followed by PCR amplified using Phusion DNA polymerase (NEB) for 15 PCR cycles. After quantified by TBS380, the paired-end RNA-seq sequencing library was sequenced with the Illumina HiSeq×TEN (2 × 150bp read length). The processing of original images to sequences, base-calling, and quality value calculations were performed using the Illumina GA Pipeline (version 1.6), in which 150bp paired-end reads were obtained. A Perl program was written to select clean reads by removing low-quality sequences, reads with more than 5% of N bases (unknown bases), and reads containing adaptor sequences. A false discovery rate (FDR) ≤ 0.001 and fold change > 2 were adopted to determine *Bacillus* sp. strain B5 differentially expressed genes between *Bacillus* sp. strain B5-only treatment and the combined treatment using DESeq2. Upregulated and downregulated differentially expressed genes were performed by GO and KEGG enrichment analysis, respectively.
